# Defective neurite elongation and branching in Nibp/Trappc9 deficient zebrafish and mice

**DOI:** 10.7150/ijbs.78489

**Published:** 2023-06-19

**Authors:** Min Hu, Brittany Bodnar, Yonggang Zhang, Fangxin Xie, Fang Li, Siying Li, Jin Zhao, Ruotong Zhao, Naveen Gedupoori, Yifan Mo, Lanyi Lin, Xue Li, Wentong Meng, Xiaofeng Yang, Hong Wang, Mary F. Barbe, Shanthi Srinivasan, John R. Bethea, Xianming Mo, Hong Xu, Wenhui Hu

**Affiliations:** 1 Laboratory of Stem Cell Biology, State Key Laboratory of Biotherapy, West China Hospital, West China Medical School, Sichuan University, Chengdu 610041, China.; 2 Center for Metabolic Disease Research, Department of Pathalogy and Laboratory Medicine, Temple University Lewis Katz School of Medicine, Philadelphia, PA, USA.; 3 Center for Stem Cell Research and Application, Institute of Blood Transfusion, Chinese Academy of Medical Sciences & Peking Union Medical College (CAMS & PUMC), Chengdu 610052, China.; 4 Department of Biology, Drexel University, Philadelphia, PA, USA.; 5 Division of Digestive Diseases, Department of Medicine, Emory University School of Medicine, Atlanta, Georgia, USA.; 6 Department of Clinical Laboratory, Xi'an NO. 3 Hospital, Xi'an, Shaanxi, 710018, China.

## Abstract

Loss of function in transport protein particles (TRAPP) links a new set of emerging genetic disorders called “TRAPPopathies”. One such disorder is NIBP syndrome, characterized by microcephaly and intellectual disability, and caused by mutations of *NIBP/TRAPPC9*, a crucial and unique member of TRAPPII. To investigate the neural cellular/molecular mechanisms underlying microcephaly, we developed Nibp/Trappc9-deficient animal models using different techniques, including morpholino knockdown and CRISPR/Cas mutation in zebrafish and Cre/LoxP-mediated gene targeting in mice. Nibp/Trappc9 deficiency impaired the stability of the TRAPPII complex at actin filaments and microtubules of neurites and growth cones. This deficiency also impaired elongation and branching of neuronal dendrites and axons, without significant effects on neurite initiation or neural cell number/types in embryonic and adult brains. The positive correlation of TRAPPII stability and neurite elongation/branching suggests a potential role for TRAPPII in regulating neurite morphology. These results provide novel genetic/molecular evidence to define patients with a type of non-syndromic autosomal recessive intellectual disability and highlight the importance of developing therapeutic approaches targeting the TRAPPII complex to cure TRAPPopathies.

## Introduction

During neuronal polarization, the initiation/extension of dendrites and axons (neuritogenesis) is followed by dendritic spine maturation (spinogenesis) and synapse formation (synaptogenesis). This dynamic process is fundamental in regulating neurogenesis (embryonic and adult), neuroregeneration, and neural plasticity. Impairment in any steps of neuronal polarization contributes significantly to a large range of neuropathologies including neuropsychiatric/neurodevelopmental disorders^1^-^6^, neurodegenerative diseases^7^-^12^, and neural injuries^13^-^15^. Although a great deal of information is available on the cytoskeletal organization, dynamic motor machinery, and signaling regulation during neuronal polarization, a large gap still exists in the understanding of the intracellular regulators and their signaling pathways^14^, ^15^. Golgi-derived protein trafficking is critical for neuronal polarization^16^-^19^, but the molecular mechanisms remain largely unknown.

Golgi trafficking-related protein NIBP/TRAPPC9 was identified by a yeast-two hybrid screen as a protein that directly binds to NIK and IKK2 proteins to form a unique NIK-NIBP-IKK2 complex that modulates the activation of the canonical and non-canonical NF-κB pathways^20^. Since then, this protein has been also identified as a homologue of yeast Trs120p, which is a unique component of the transport protein particle (TRAPP) II complex^21^, ^22^. Like its yeast counterpart, the mammalian TRAPPII complex is associated with COPI-coated vesicular structures and is likely to function in vesicle tethering and transport within the early Golgi and from endosome to *trans*-Golgi to regulate Golgi trafficking^21^, ^23^. Thus, Nibp/Trappc9 protein is involved in regulating the functions of NF-κB pathways and vesicle trafficking in cells^24^, ^25^. These two fundamental biologic processes and their interactions may play essential roles in regulating neuronal polarization.

An increasing number of genome-wide association studies have identified an emerging set of genetic disorders called “TRAPPopathies” that link to variations and mutations in TRAPP-associated proteins^26^, ^27^. These variations cause distinct disorders with overlapping phenotypes, but the underlying mechanisms remain elusive. Mutations in *TRAPPC9* are associated with non-syndromic autosomal recessive intellectual disability (NS-ARID), assigned as “Intellectual Disability-Obesity-Brain Malformations-Facial Dysmorphism Syndrome” and “Intellectual developmental disorder, autosomal recessive 13” (OMIM #613192), and abbreviated as NIBP syndrome^24^, ^28^. Loss-of-function *TRAPPC9* mutations manifest microcephaly, intellectual disability, and obesity in patients^29^-^45^. Magnetic resonance imaging in affected individuals with NIBP syndrome has revealed reduced cerebral white matter volume with sulcal enlargement, thinning of the corpus callosum, and mild cerebellar volume loss^29^-^31^. Trappc9 (mTrappc9) null mice show a reduction in brain size, exploratory activities, and social memory, as well as a marked increase in body weight^34^, ^46^. Furthermore, an imbalance is found between dopamine D1 and D2 receptor-containing neurons in the brain of mTrappc9-deficient mice^46^. However, the mechanisms of how TRAPPC9 deficiency causes the neural phenotypes including microcephaly and intellectual disabilities in patients remains unknown. Here, we used zebrafish and mouse models to illuminate the neural cellular and molecular mechanisms underlying microencephaly involved in NIBP syndrome. We found that both models recapitulated neurodevelopmental phenotypes in patients with NIBP syndrome. Trappc9 deficiency did not influence the number or type of neural cells during embryonic and adult neurogenesis as well as neurite initiation process. However, Trappc9 dysfunctions in zebrafish and mice impaired the neurite elongation and branching processes. These findings highlight the exploration of novel therapeutic targets on TRAPPII trafficking to cure TRAPPopathies.

## Results

### Trappc9 deficiency in zebrafish and mice causes microcephaly and intellectual disability, similar to NIBP syndrome patients

To elucidate the role of Trappc9 during early neurodevelopment, we chose to use a zebrafish model. We designed two different antisense morpholino oligonucleotides (MO1, MO2) (Figure S1A) that target zebrafish *Trappc9* (z*Trappc9)* RNA to knock down z*Trappc9* expression. Knockdown of z*Trappc9* mRNA in zebrafish embryos was confirmed by RT-qPCR (Figure S1B, C). Through real-time observation, we found that embryos injected with MO1 or MO2 at the 1-cell stage had visibly decreased brain size at 48- and 72-hour post fertilization (hpf) (Figure S2A, B). To further confirm these results, we used CRISPR/Cas9 technology to generate five separate homozygous mutant lines with the disruption of z*Trappc9* (Figure S1D, E). All five homozygous mutants were effective at decreasing z*Trappc9* expression, with any adult and embryonic zebrafish displaying smaller brains (Figure S2A-D). These results indicate that zTrappc9 deficiency can cause microcephaly in zebrafish. Strikingly, around 83% of the homozygous mutant 1-cell stage embryos remained undivided and failed to undergo further development (Figure S1F).

To further recapitulate the neurodevelopmental phenotype of patients with NIBP syndrome, we generated *mTrappc9* floxed transgenic mice using a traditional gene targeting strategy (Figure S1G). Crossbreeding with germline deletion Ella-Cre mice resulted in global knock-out mice with deletion of exons 2-5 within the *mTrappc9* gene, resulting in loss-of-function for both isoform I (960 aa) and II (1139 aa) of mouse *mTrappc9*. These mice carried *mTrappc9* C-terminal mutant mRNA that continued to translate a truncated Trappc9 protein containing C-terminal sequence (Figure S1G, H, J-L). The truncated Trappc9 protein was able to form complexes with Trappc10 protein (Figure S1K). Homozygous *mTrappc9* knockout (*mTrappc9^m/m^*) mice displayed no significant difference in neonatal brain size but presented significant reduction in the size/volume at postnatal day 20 (P20) and adult stages compared to wildtype littermates (Figure S2E-H, K, L)*.* In addition, we noted smaller spinal cords in *mTrappc9^m/m^* mice (Figure S2I, J). To assess the functional impact of brain/spinal cord defects, we performed general behavior and neurobehavior tests as previously described^47^. We did not observe any significant differences between wildtype and *mTrappc9^ m/m^* mice for most of the tests, including general behaviors (motor, sensory and nociceptive), olfactory function, cold plate test, marble burying, and nestling (data not shown). However, we did find differences in exploratory activity and memory. In an Open Field (OF) test, adult *mTrappc9^ m/m^* mice moved less than their wildtype littermates. We also performed a novel objective test, a contextual fear test, and a Barns maze test. All the tests showed that *mTrappc9^m/m^* mice expressed decreased explorative activity and an impaired memory (Figure S3)^34^, ^46^.

Besides microcephaly and some behavioral differences, our pipeline screening of *mTrappc9^ m/m^* mice found a significant and steady increase in body weight compared to wildtype littermates (Figure S1I). Additionally, male *mTrappc9^ m/m^* mice were infertile (data not shown). Taken together, the anatomic phenotypes observed in Trappc9 deficient zebrafish and mice are consistent with previous observations made by others working with mTrappc9 deficient mice targeting exon-5 or exon-7^27^, ^34^, ^46^ and phenotypical characteristics of patients with NIBP syndrome^31^.

### Both grey and white matter volumes decrease in Trappc9 deficient zebrafish and mouse brains

One possible cause of microcephaly may be due to impaired neurogenesis, leading to a reduced number of neural cells and/or insufficient neurites and synaptic connections. Neurogenesis (the generation and maturation of neural cells) occurs not only during embryonic and perinatal development, but also in adult nervous system^3^, ^48^-^52^. To understand how Trappc9 deficiency may cause microcephaly, we first looked at embryonic neurogenesis in zebrafish using whole mount *in situ* hybridization (WISH) to examine the changes in neural stem cells (NSCs) and neural progenitor cells (NPCs) with the well-established marker *sox2*. There was no significant difference in *sox2* expression patterns or levels between *zTrappc9* mutant, *zTrappc9* morphant, and wildtype embryos at 24 hpf, 48 hpf (data not shown), or 72 hpf (Figure S4A, B).

We next examined the expression of cellular markers for other neural cell types using *elavl3* (pan-neuronal marker), *olig2* (oligodendrocyte marker), *slc1a3a* (*glast*, astrocyte marker), and gfap (radial glia-astrocyte lineage marker) in zebrafish embryos; however, we did not detect any remarkable difference in the expression pattern and intensity among any groups (data not shown). Meanwhile, we examined the development of neural cells in mouse embryos by immunofluorescence staining. The expression of PAX6/SOX2 (neuroepithelia and NSCs/NPCs), TBR2 (NPCs), and LHX2/BCL11B (neurons) showed no alterations in the embryonic brains of mTrappc9^ m/m^ mice (Figure S4C-H). Similarly, the spatial structures and the arrangements of neurons, oligodendrocytes, astrocytes, and microglia were not altered in the cerebral cortex and/or spinal cord of adult mTrappc9^ m/m^ mice compared to wildtype mice (Figure S4I-V). Taken together, these observations demonstrate that Trappc9 deficiency is unlikely able to modulate embryonic neurogenesis and development of several neural cell types.

**Figure 1:**
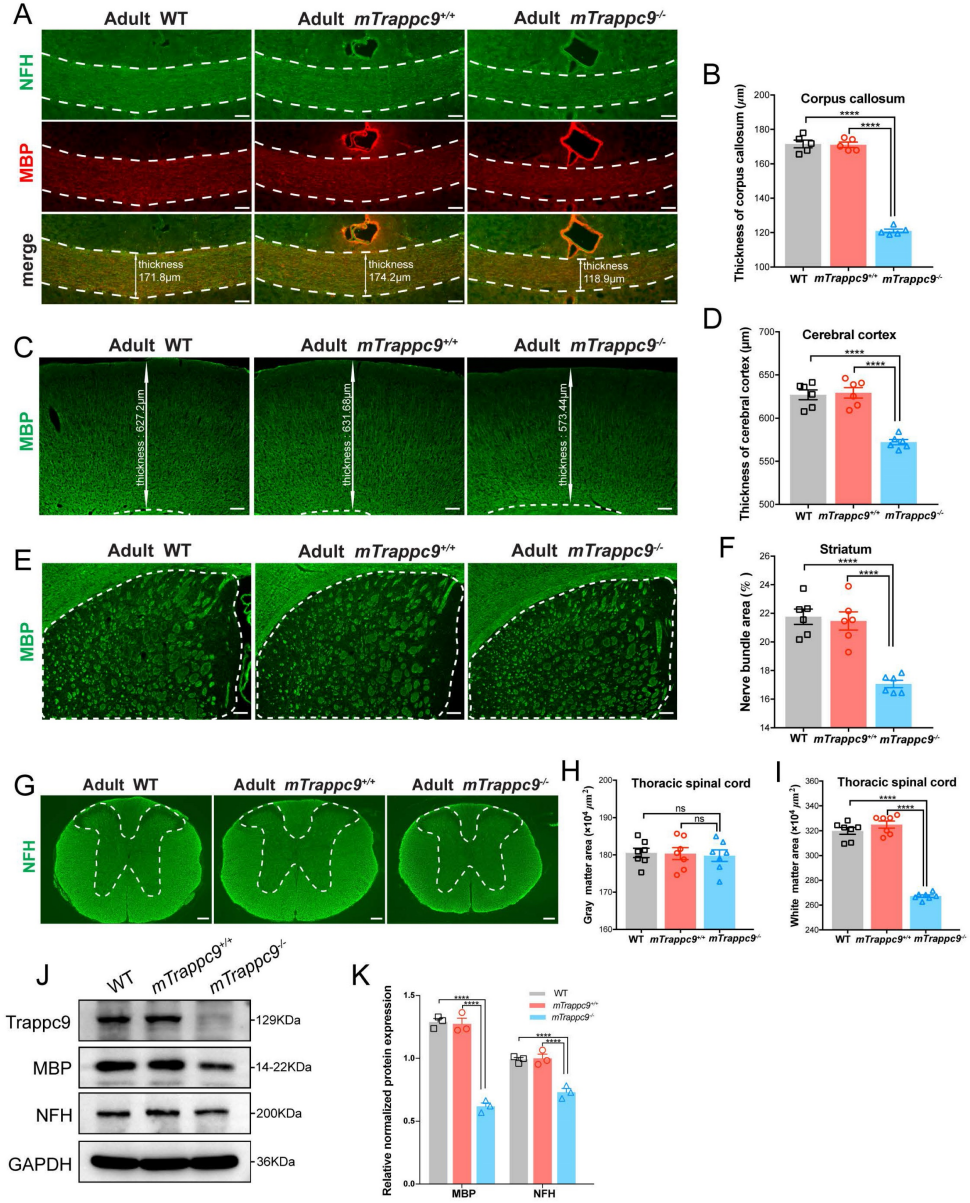
** White matter and cerebral cortex are reduced in adult *mTrappc9* deficient mice. (A-B)**, NFH (axonal tracts) and MBP (myelin) immunostaining of the corpus callosum sections. Compared with WT (C57BL/6 wildtype) and *mTrappc9^+/+^*, the corpus callosum (highlighted with white dotted lines) of *mTrappc9^m/m^* mice was significantly thinner. Representative images with indicated thickness from *n*=3 mice/group (**A**), and quantitative analysis of *n*=5 sections (**B**). **(C-D)**, MBP immunostaining of the cerebral cortex sections, with the white line arrows defining the representative thickness of the cerebral cortex (**C**, *n*=3 mice). Quantification shows the cortex is significantly thinner in *mTrappc9^ m/m^* mice than the other two groups (**D**, *n*=6 sections). **(E-F)**, MBP immunostaining of the striatum. The white dotted lines demarcate the striatum area (**E**, *n*=3 mice) for quantification of the coverage area of the nerve bundles in the striatum (**F**, *n*=6 sections), which shows that nerve bundles are significantly reduced in *mTrappc9^ m/m^* mice compared to WT and *mTrappc9^+/+^* mice. **(G-I)**, NFH immunostaining of spinal cord sections. In *mTrappc9^ m/m^* mice, there was no significant difference in gray matter (inside the white dotted line), but the area of white matter (outside the white dotted line) was reduced significantly. Representative images from *n*=3 mice/group (**G**); *n*=7 sections (**H, I**). **(J-K)**, Western blot analysis of MBP and NFH protein expression in cerebral cortex normalized with GAPDH. Both showed a significant decrease in *mTrappc9^ m/m^* mice. *n*=3 mice/group. Data are means ± SEM; One-way ANOVA (**B, D, F, H, I**); Two-way ANOVA (**K**); ****P≤0.0001; ns, not significant (P>0.05). Scale bars: 50 μm (**A**); 150 μm (**C, E, G**).

During our immunofluorescence analysis, we noticed reduced grey and white matter volumes in brains and spine cords of adult *mTrappc9^ m/m^* mice. We therefore immunostained the brains and spinal cords of adult *mTrappc9^m/m^* mice with anti-neurofilament heavy chain (NFH, nerve fiber marker) and anti-myelin basic protein (MBP, myelin marker) antibodies. Both NFH and MBP immunostainings can show clear identification of white matter. We confirmed a decrease in thickness of the corpus callosum and cerebral cortex (Figure 1A-D), as wells as significant reduction of white matter in the striatum and thoracic spinal cord in the brains of *mTrappc9^m/m^* mice (Figure 1E-I). Western blot analysis using whole brain lysates from *mTrappc9^ m/m^* mice also showed decrease of NFH and MBP protein expression (Figure 1J, K). These results suggest that the gross volume reduction of brain and spinal cord caused by Trappc9 deficiency predominantly occurs as a direct consequence of the decrease in grey and white matter sizes (areas) in animal brains.

### Neurite development defects in brains of Trappc9 deficient zebrafish and mice

Brain white matter mainly consists of axons, myelin, astrocytes, oligodendrocytes, and microglia whereas brain grey matter mainly consists of axons, dendrites, and bodies of neurons.

**Figure 2:**
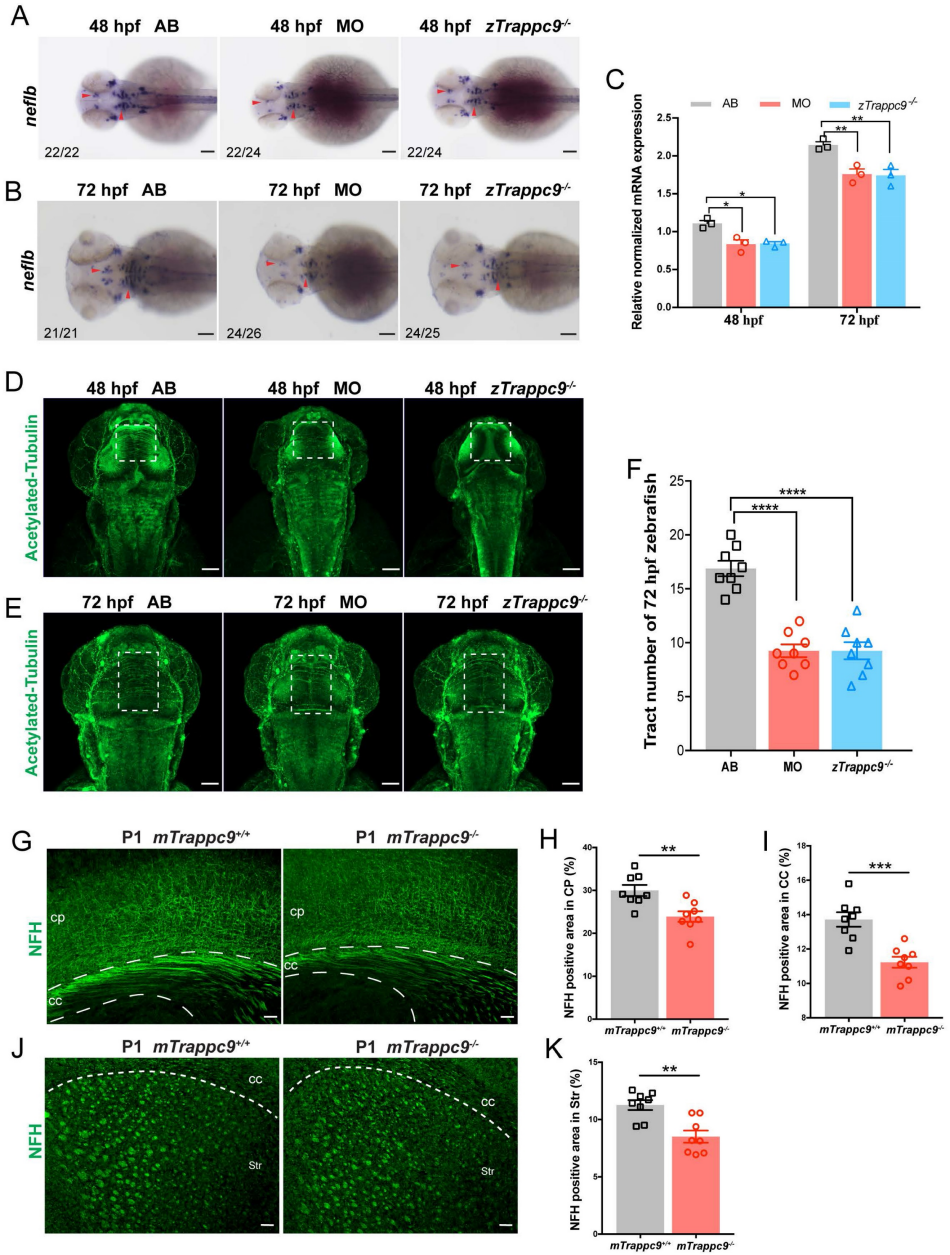
** The axonal tracts of early developing mice and zebrafish are reduced after *Trappc9* loss-of-function. (A-C)**, RNA *in situ* hybridization and RT-qPCR analysis of *neflb* mRNA expression in mutant (*zTrappc9^m/m^*) and morphant (MO) zebrafish embryos at 48 and 72 hpf. The red arrowheads point to the expression of *neflb* in the midbrain and hindbrain of zebrafish embryos. Both showed a decrease of *neflb* mRNA in MO and *zTrappc9^m/m^* zebrafish embryos. Representative images from *n*=20-26 embryos/group in 7 experiments (**A, B**); *n*=3 experiments (**C**). **(D-F)**, Acetylated-α-tubulin immunostaining of zebrafish embryos at 48 and 72 hpf to label axonal tracts. The dotted square lines indicate intertectal fascicles and commissures. (**F**) Statistical analysis of the number of internal fascicles and commissure of zebrafish at 72 hpf in (**E**). The number of axonal tracts in MO and *zTrappc9^m/m^* zebrafish were significantly reduced compared to wildtype AB line. Representative images from *n*=10 embryos/group in 4 experiments (**D, E**); *n*= 8 embryos (**F**). **(G-K)**, NFH immunostaining of neonatal mouse cerebral cortex (CP)/corpus callosum (CC), and striatum (Str). The axonal tracts of *mTrappc9^m/m^* mice were significantly fewer than *mTrappc9^+/+^* mice in CP (**H**), CC (**I**) and Str (**K**). Representative images from* n*=4 mice/group (**G, J**); *n*=8 sections (**H, I, K**). Data are means ± SEM; *t*-tests (**H, I, K**); One-way ANOVA (**F**); Two-way ANOVA (**C**); ****P≤0.0001; ***P≤0.001; **P≤0.01; *P≤0.05; Scale bars: 50 μm (**A, B, D, E, G, J**).

Since we found no apparent differences in several neural cell types in the brains of Trappc9 deficient zebrafish or mice, we decided to examine the embryonic development of axons in zebrafish. WISH experiments showed that the expression of neurofilament light chain B (*neflb*) gene, which is responsible for axonal growth, was significantly decreased in the brains of z*Trappc9* mutant and morphant zebrafish at embryonic stages 48 hpf and 72 hpf (Figure 2A-C). Immunofluorescence staining with anti-acetylated tubulin showed fewer/shorter axons in the embryonic brains of z*Trappc9* mutant and morphant zebrafish (Figure 2D-F). As the expression of myelin genes occurs after 60 hpf, these results indicate that axon development is defective prior to myelination. On the other hand, staining of newborn and adult mouse brains with anti-NFH showed dramatically reduced axons in the cerebral cortex, the striatum, and the corpus callosum in *mTrappc9^m/m^* mice (Figure 2G-K). Taken together, the results show that Trappc9 deficiency reduces axon generation of neurons in the brains during embryonic development.

**Figure 3:**
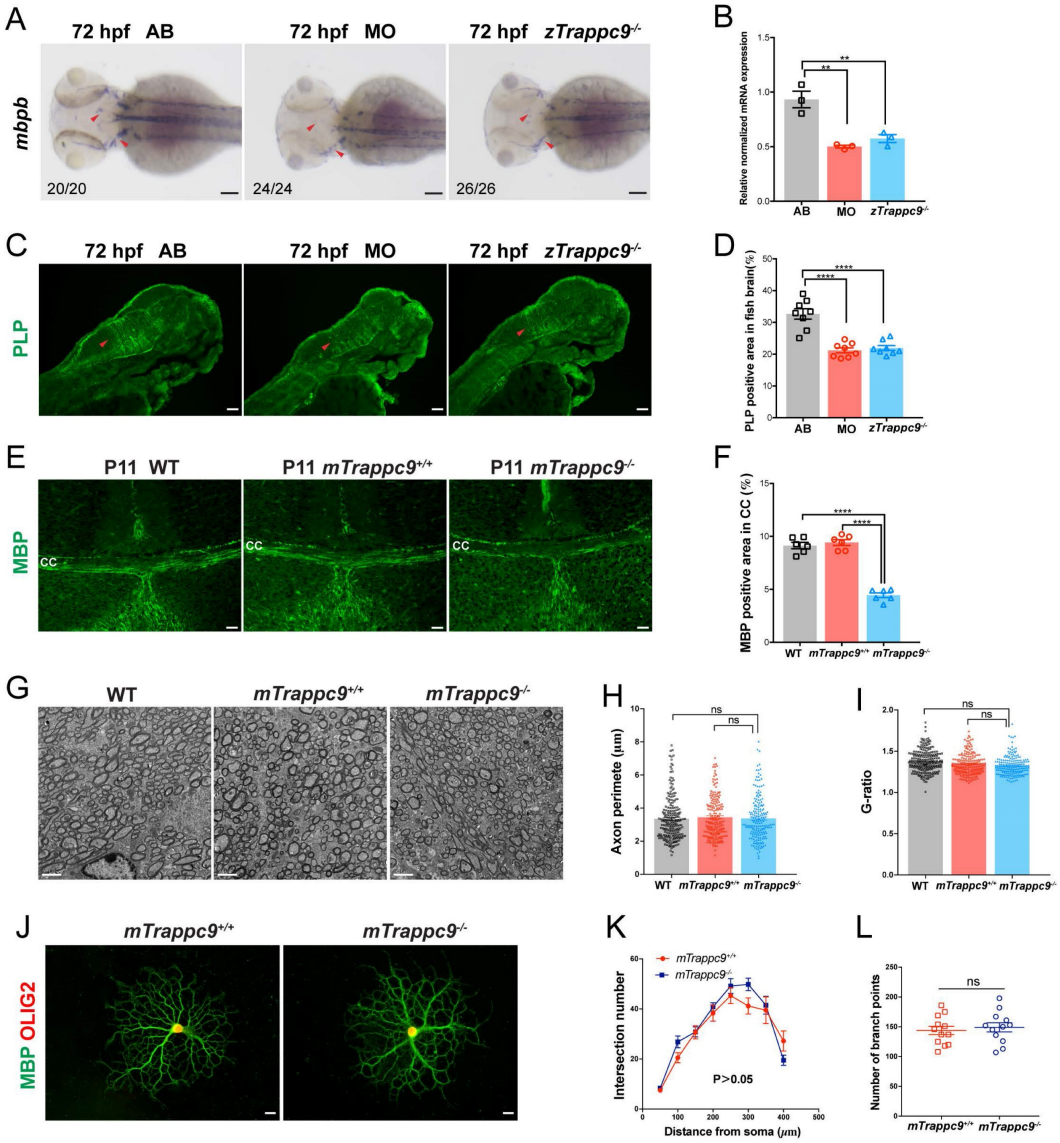
** Myelin sheath in early developing mice and zebrafish is reduced after *Trappc9* deficiency, but the myelin sheath formation was not affected. (A-B)**, RNA *in situ* hybridization and RT-qPCR analysis of *mbpb* expression in 72 hpf zebrafish embryos. The expression of *mbpb* in mutant (*zTrappc9^m/m^*) and morphant (MO) zebrafish at 72 hpf was significantly reduced. Representative images from *n*=20-26 embryos/group in 7 experiments (**A**); *n*=3 experiments (**B**). **(C-D)**, PLP immunostaining of 72 hpf zebrafish embryos. The area percentage of PLP fluorescence in zebrafish hindbrain (red arrowheads) was significantly decreased in MO and *zTrappc9^m/m^* zebrafish embryos. Representative images from *n*=10 embryos/group (**C**); *n*=8 sections (**D**). **(E-F)**, MBP immunostaining of P11 mouse cerebral cortex, showing the area percentage of MBP fluorescence in the corpus callosum was significantly decreased in *mTrappc9^m/m^* mice. Representative images from *n*=3 mice/group I; *n*=6 sections (**F**). **(G-I)**, Transmission electron microscope (TEM) micrographs of adult mouse corpus callosum showing no significant difference in the axon perimeter (**H**) and G-ratio (**I**) in *mTrappc9^m/m^* mice. Representative images from *n*=13 sections/group (**G**); *n*=200-220 myelinated axons (**H, I**). **(J-L)**, MBP and OLIG2 immunostaining of primary cultured oligodendrocytes, which were differentiated from brain-derived oligodendrocyte precursor cells (OPCs) of P8 mice. The morphology (sholl analysis, **K**) and branch numbers (**L**) of oligodendrocytes were not affected by *mTrappc9* deficiency. Representative images from *n*=3 coverslips/group (**J**); *n*=6 neurons (**K**); *n*=12 neurons (**L**). Data are means ± SEM; *t*-tests (**L**); One-way ANOVA (**B, D, F, H, I**); Two-way ANOVA (**K**); ****P≤0.0001; **P≤0.01; ns, not significant (P>0.05). Scale bars: 2 μm (**G**); 10 μm (J); 50 μm (**A, C, E**).

Next, we examined the development of myelin in embryonic brains of zebrafish. WISH experiments showed significantly decreased expression of *mbpb* (a well-established marker for myelin) in the brains of *zTrappc9* mutant and morphant zebrafish at 72 hpf (Figure 3A, B). Immunostaining with anti-PLP (myelin marker) showed robust reduction of myelin at 72 hpf in the brains of the *zTrappc9* mutant and morphant zebrafish (Figure 3C, D). Similarly, we observed dramatic reduction of MBP^+^ myelin in the striatum in brains from *mTrappc9^m/m^* mice at stage P11 (Figure 3E, F), when myelination typically occurs in the developing mouse brain^53^. However, ultrastructure imaging of the corpus callosum of *mTrappc9^m/m^* mouse brains did not show any significant alternation in the multilayered compact myelinated structures (Figure 3G, I). Additionally, axons also did not show any morphological abnormalities (Figure 3G, H). To further validate these findings, we cultured oligodendrocyte precursor cells (OPCs) and differentiated them into oligodendrocytes. In agreement with the ultrastructural experiments, oligodendrocytes from *mTrappc9^m/m^* mice did not exhibit any morphologic abnormalities compared to those from wildtype mice (Figure 3J-L). These results suggest that Tracpp9 deficiency does not impair the structure of myelin sheaths, although the number and density of myelin sheaths are dramatically reduced. This suggests that the reduction of myelin is a secondary effect due to decreased axons in Trappc9 deficient brains.

**Figure 4:**
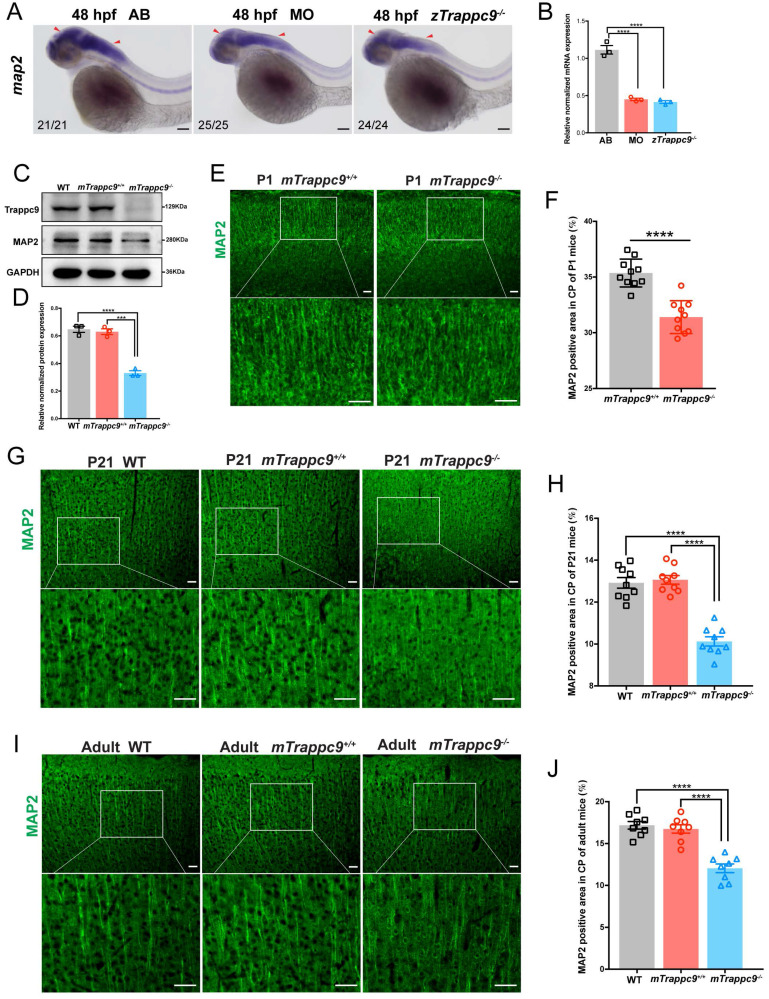
** Dendrites of neurons are reduced after *Trappc9* loss-of-function. (A-B)**, RNA *in situ* hybridization and RT-qPCR analysis of *map2* mRNA expression in 48 hpf zebrafish embryos. The expression of *map2* was reduced in mutant (*zTrappc9^m/m^*) and morphant (MO) zebrafish embryos. Representative images from *n*=20-25 embryos/group in 5 experiments (**A**); *n*=3 experiments (**B**). **(C-D)**, Western blot analysis of MAP2 in adult mouse cerebral cortex showing significant reduction in the integrated density of MAP2 protein bands normalized with GAPDH. *n*=3 mice/group. **(E-F)**, MAP2 immunostaining of neonatal (P1) mouse cerebral cortex showing a significant decrease in the area ratio of MAP2 fluorescence. Representative images from *n*=3 mice/group (**E**); *n*=10 sections (**F**). **(G-H)**, MAP2 immunostaining of P21 mouse cerebral cortex showing a significant decrease in the area ratio of MAP2 fluorescence. Representative images from *n*=3 mice/group (**G**); *n*=9 sections (**H**). **(I-J)**, MAP2 immunostaining of adult mouse cerebral cortex showing a significant decrease in the area ratio of MAP2 fluorescence. Representative images from *n*=3 mice/group (**I**); *n*=8 sections (**J**). Data are means ± SEM; *t*-tests (**F**); One-way ANOVA (**B, D, H, J**); ****P≤0.0001; ***P≤0.001; Scale bars: 50 μm (**A, E, G, I**).

We next focused on the development of dendrites as well as synapses in the brains of animals. WISH experiments on *map2* expression showed that dendrites were dramatically reduced in embryonic *zTrappc9* mutant and morphant zebrafish brains (Figure 4A, B). Immunostaining using anti-Map2 antibody showed reduction of dendrites in the cerebral cortex of *mTrappc9^m/m^* P1, P21, and adult mouse brains (Figure 4C-J), indicating that Trappc9 modulates the dendrite development of neurons. Furthermore, immunostaining with the synaptic markers anti-synapsin (SYN), synaptophysin (SYP), PSD95, or synaptotagmin-2 (SYT2) showed that Trappc9 deficiency significantly reduced the presynaptic and postsynaptic densities in the cerebral cortex of *mTrappc9^m/m^* mice (Figure S5A-H). Thus, Trappc9 deficiency impairs axons, dendrites, and synapses during neurodevelopment in animal brains.

### Trappc9 maintains TRAPPII complex in brain neurons

To ascertain how Trappc9 deficiency vitiated neurites, we performed single-cell RNA sequencing (scRNA-seq) and analyzed the types of neural cells in the cerebral cortex of newborn mice. In agreement with previous findings, these results showed that Trappc9 deficiency did not change the cellular types in the cerebral cortex (Figure S6A, B). Gene expression profiling showed that most genes with altered expression were primarily related to neuritogenesis, spinogenesis, synaptogenesis, and vesicle trafficking (Figure S6C, D). Given that Nibp/Trappc9 regulates canonical and non-canonical pathways of NF-κB activation^20^, we examined the expression modalities of NF-κB targeted genes in zebrafish embryonic brains. However, the results showed no significant changes in the expression of NF-κB targeted genes (Figure S6E), suggesting that Trappc9 modulation of neurite development likely occurs via an alternative NF-κB-unrelated mechanism, consistent with previous observations^46^.

Next, we performed mass spectrometry-based proteomics using isolated proteins from adult mouse cerebral cortex. Interestingly, we found that several proteins, including components of TRAPPII complex^54^, Trappc1, Trappc2l, Trappc3, Trappc4, Trappc5, Trappc6b, and Trappc10, possessed lower expression in *mTrappc9^m/m^* mice (Figure S6F).

We also found decreased expression of proteins involved in axonogenesis, neuronal projection extension, and vesicle targeting, cycling, and transport (Figure S6G, H). TRAPPII complex has been shown to participate in protein trafficking to Golgi and endosomes and binds to COPI-coated vesicles^21^, ^22^. Vesicle trafficking is a key factor in modulating neuritogenesis, spinogenesis, and synaptogenesis during embryonic and adult neurogenesis. As scRNA-seq transcriptomics and proteomics suggested altered vesicle trafficking in neurons in *mTrappc9^m/m^* brains, we performed multilabeled immunocytochemical staining with antibodies against subcellular organelles (Figure S7). In neurons from wildtype *mTrappc9^+/+^* mice, either mTrappc9 or mTrappc10 was colocalized with GM130 (Golgi apparatus) and COPG (COPI). However, we did not observe any significantly visible alterations in the morphologic appearances, structures, and distributions of Golgi apparatuses, endosomes, and COPI-coated vesicles in neurons derived from the brains of *mTrappc9^m/m^* mice compared to wildtype littermates (Figure S7).

**Figure 5:**
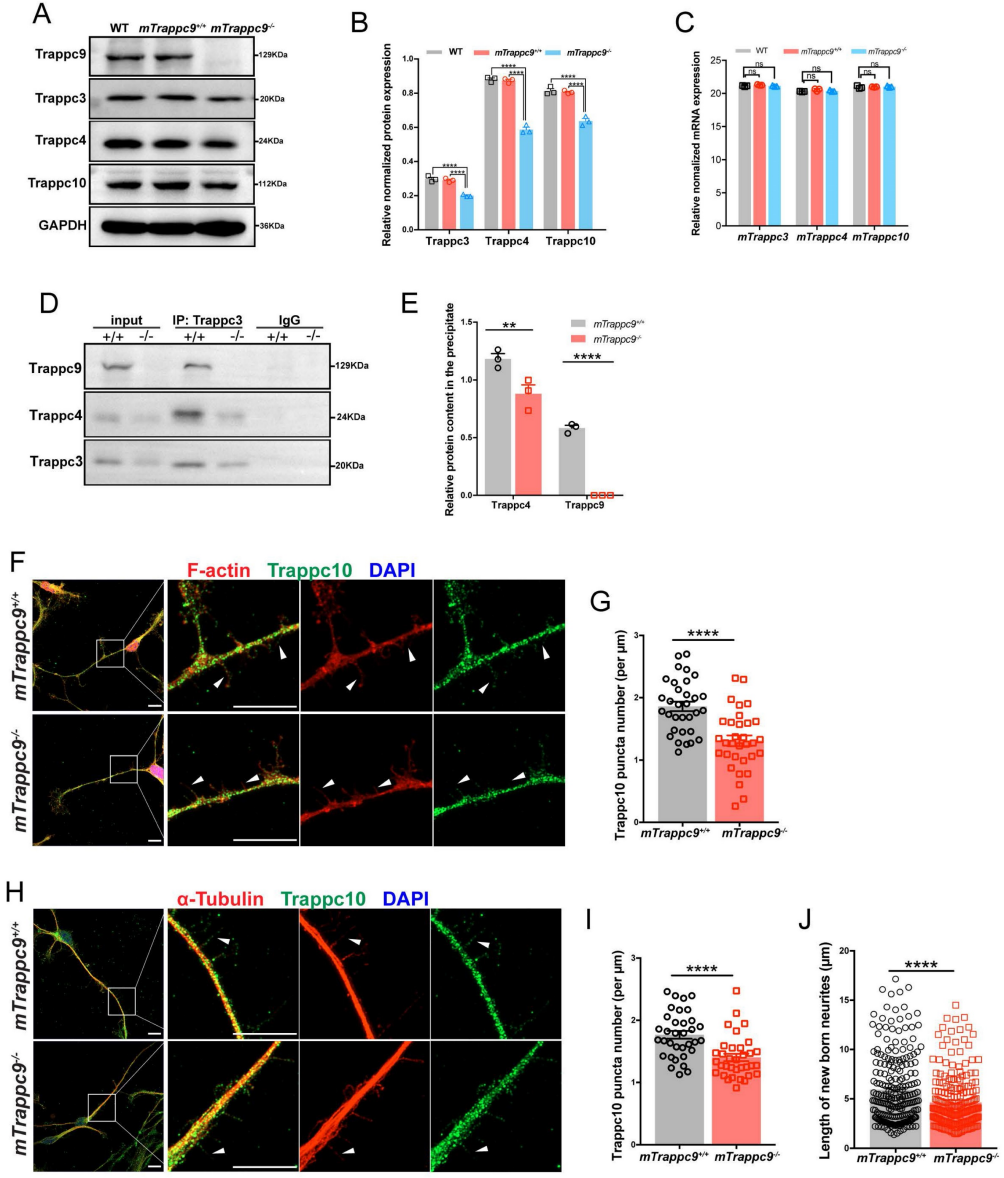
** The distribution of Trappc10 along microfilaments and microtubules is reduced after *mTrappc9* deficiency. (A, B)**, Western blot analysis of mTrappc3/4/9/10 in adult mouse cerebral cortex showing a significant decrease in the integrated density of protein bands normalized with GAPDH. *n*=3 mice/group. **(C)**, RT-qPCR analysis of *Trappc3*, *Trappc4*, *Trappc10* showing no significant differences.* n*=3 mice/group. **(D, E)**, Co-immunoprecipitation with Trappc3 antibody. Trappc3 can pull down full-length mTrappc9 in the brain tissue lysate from *mTrappc9^+/+^* mice, but not *mTrappc9^ m/m^* mice. Trappc4 can be co-immunoprecipitated by Trappc3 in *mTrappc9^+/+^* mice but was significantly reduced in the co-precipitate of *mTrappc9^ m/m^* mice. *n*=3 mice/group. Quantitative data represent the ratio of precipitated protein over corresponding input for normalization. **(F-I)**, Trappc10 and F-actin/α-tubulin immunostaining of mouse NSC-differentiated neurons cultured for 6 days showing significant decrease in the Trappc10 puncta number per micron of nascent neurites. Arrowheads indicated several nascent neurites, which showed the distribution of Trappc10 reduced in *mTrappc9^m/m^* neurons compared to *mTrappc9^+/+^*. Representative images from *n*=8 coverslips/group (**F, H**); *n*=32 nascent neurites (**G, I**). **(J)**, Statistics of the length of nascent neurites in *mTrappc9^+/+^* and *mTrappc9^m/m^* neurons. The nascent neurites of *mTrappc9^m/m^* are shorter than *mTrappc9^+/+^*. *n*=231 nascent neurites. Data are means ± SEM; t-tests (**G, I, J**); Two-way ANOVA (**B, C, E**); ****P≤0.0001; **P≤0.01; ns, not significant (P>0.05). Scale bars: 10 μm (**F, H**).

We validated the proteomic data by western blot analysis, which showed that expression of TRAPPII complex components were largely decreased in the cerebral cortex of *mTrappc9^m/m^* mouse brains (Figure 5A, B), although, RT-qPCR analysis did not show significant changes in mRNA levels of these markers (Figure 5C). These results suggest that mTrappc9 deficiency causes the instability of TRAPPII complexes or their individual components in neurons of the cerebral cortex. We carried out co-immunoprecipitation (co-IP) to further verify the instability of TRAPPII complex in *mTrappc9^m/m^* mice and found that mTrappc3 pulled down less mTrappc4 than in littermate *mTrappc9^+/+^* mice (Figure 5D, E). Since mTrappc10 is another unique component of TRAPPII, we performed multilabeled immunofluorescence staining to evaluate the distribution and status of mTrappc9 and mTrappc10 in neurons differentiated from NSCs/NPCs derived from E13.5 embryonic brains of *mTrappc9^+/+^* and *mTrappc9^m/m^* mice. As predicted, mTrappc9 displayed typical punctate foci in the cytoplasm, growth cones, and neurites (dendrites and axons) of neurons, with mTrappc10 showing a similar distribution. In addition, many of the mTrappc9^+^ and mTrappc10^+^ puncta were distributed along F-actin and microtubules in growth cones and neurites (Figure 5F-J, S8). In *mTrappc9^m/m^* mice, the punctate distribution of mTrappc9 immunoreactivity remained detectable (Figure S8), because a truncated protein (787 aa, 87.4 kDa) translated at the first translation initial site (ATG) exists as validated by co-IP and western blot analysis (Figure S1G). However, the expression level of the truncated mTrappc9 protein was much lower than the littermate control mice (Figure S8A-F). In contrast, mTrappc10^+^ puncta did not show significant alteration in the cell body of neurons (Figure S8A, B). However, there was a significant loss of mTrappc10^+^ and mTrappc9^+^ puncta along the microfilaments and microtubules of neurites of *mTrappc9^m/m^* neurons (Figure 5F-I, Figure S8C-F). We further explored the relationship between TRAPPII and microtubules/microfilaments by using latrunculin and vinblastine, which inhibit the polymerization of microfilaments and microtubules respectively and inhibit the development of neurites. As a result of this growth inhibition, the distribution of mTrappc9^+^ and mTrappc10^+^ puncta along microfilaments or microtubules decreased, similar to the phenotype of mTrappc9 deficient neurons (Figure S9), indicating that mTrappc9/mTrappc10 are associated with microtubules and/or microfilaments. These results suggest that Trappc9 plays an essential role in maintaining the stability and distribution of TRAPPII complexes in neurites as well as modulate the growth of newborn neurites.

### Reduced TRAPPII complex impairs the elongation and branching of neuronal dendrites and axons in brains of mice

To address how TRAPPII complex controls the morphogenesis of neurites, we cultured NSCs/NPCs derived from E13.5 embryonic brains of *mTrappc9^+/+^* and *mTrappc9^m/m^* mice and induced their neuronal differentiation. We first measured the length of newborn neurites of *mTrappc9^+/+^* and *mTrappc9^m/m^* neurons and found that the length of newborn neurites of *mTrappc9^m/m^* was much shorter, in comparison to those of littermate *mTrappc9^+/+^* neurons (Figure 5J), suggesting that TRAPPII complex is involved in neurite growth. Neurite growth is led by an amoeboid-like cytoplasmic enlargement at the tip, termed the growth cone, that is composed of motile sheet-like lamellipodia and narrow filopodia. To determine the features of growth cones during neurite development, we measured the F-actin protruding area on the entire cell by subtracting the area of microtubule signal from the F-actin area^55^, ^56^.

**Figure 6:**
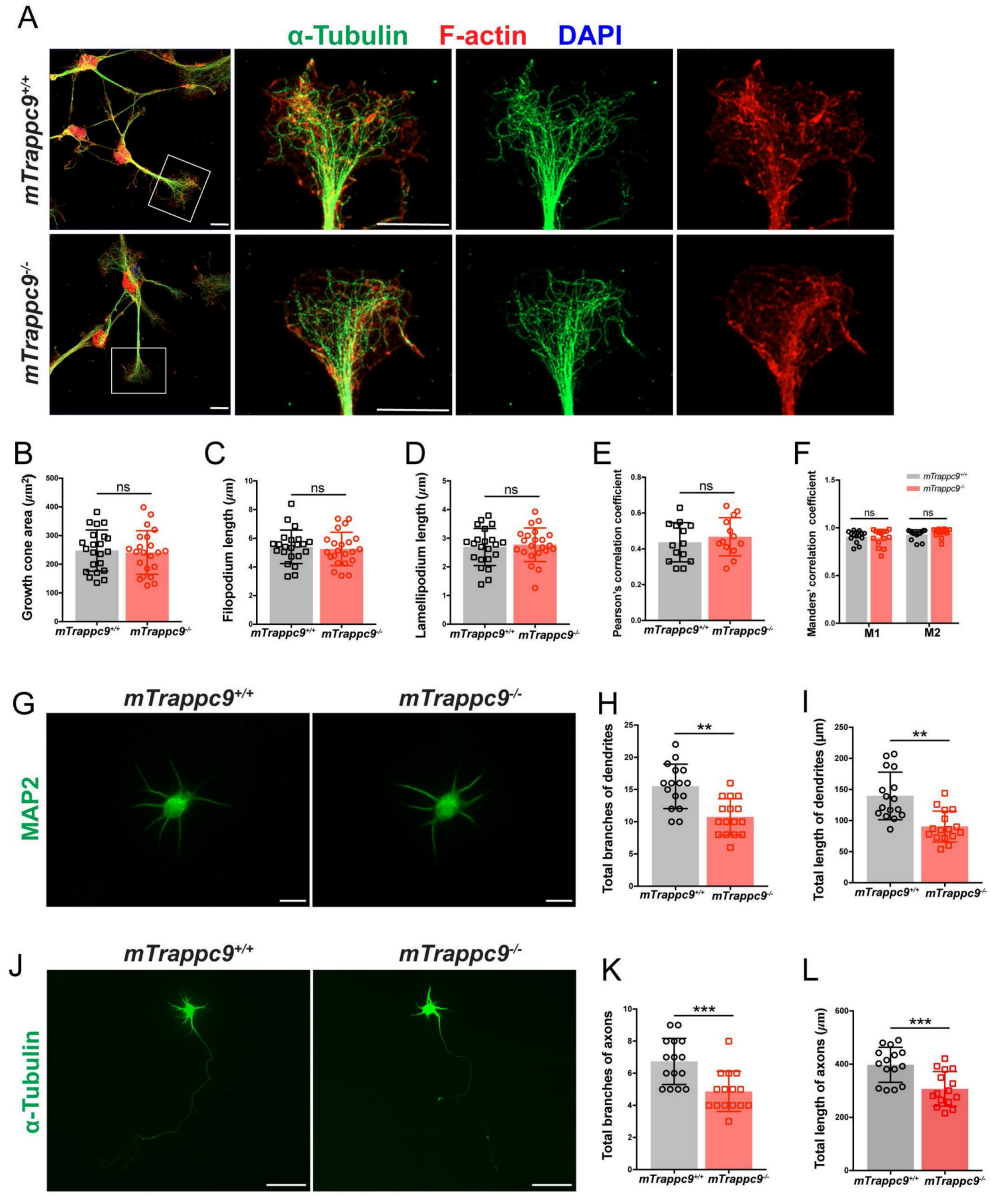
** The length and branches of nerve tracts are reduced after *Trappc9* deficiency *in vitro*. (A-F)**, F-actin and α-tubulin immunostaining of mouse NSC-differentiated neurons cultured for 6 days. (**B**) Growth cone size; (**C**) length of filopodium; (**D**) length of lamellipodium; (**E**) /(**F**) Pearson's/Manders' correlation coefficient of F-actin and α-tubulin in growth cone, respectively. *mTrappc9^m/m^* mouse neuronal growth cones had normal morphology. Representative images from *n*=3 coverslips/group (**A**); *n*=22 growth cones (**B-D**); *n*=14 growth cones (**E-F**). **(G-I)**, Dendrites of primary neurons from E17.5 mouse cerebral cortex cultured for 6 days labeled by MAP2. Both total branch points (**H**) and total lengths (**I**) of dendrites were significantly reduced in *mTrappc9^m/m^* neurons. Representative images from *n*=4 coverslips/group (**G**); *n*=16 neurons (**H, I**). **(J-L)**, Axons of primary neurons from E17.5 mouse cerebral cortex cultured for 6 days labeled by α-tubulin. Both total branch points (**K**) and total length (**L**) of axons were significantly reduced in *mTrappc9^m/m^* neurons. Representative images from *n*=4 coverslips/group (**J**); *n*=15 neurons (**K, L**). Data are means ± SEM; *t*-tests (**B, C, D, E, H, I, K, L**); Two-way ANOVA (**F**); ***P≤0.001; **P≤0.01; ns, not significant (P>0.05). Scale bars: 10 μm (**A, G**); 50 μm (**J**).

We did not observe a perturbation of the global actin protruding area in mTrappc9 deficient neurons (Figure 6A). We also looked at the growth cone morphology by analyzing the area, filopodium/lamellipodium length, and correlation coefficient of the major growth cone, but did not observe any significant changes (Figure 6B-F), indicating that mTrappc9 may not be involved in the initiation of neurite growth. Next, we cultured primary neurons from the cerebral cortex of E17.5 mouse embryonic brains and analyzed their axonal and dendritic morphologies. In comparison to wildtype littermate neurons, mTrappc9 deficient neurons had shorter dendrites with fewer branches (Figure 6G-I). The number of axonal branches (Figure 6J, K) as well as the total length of axons from the mTrappc9 deficient neurons were also significantly reduced (Figure 6J, L).

**Figure 7:**
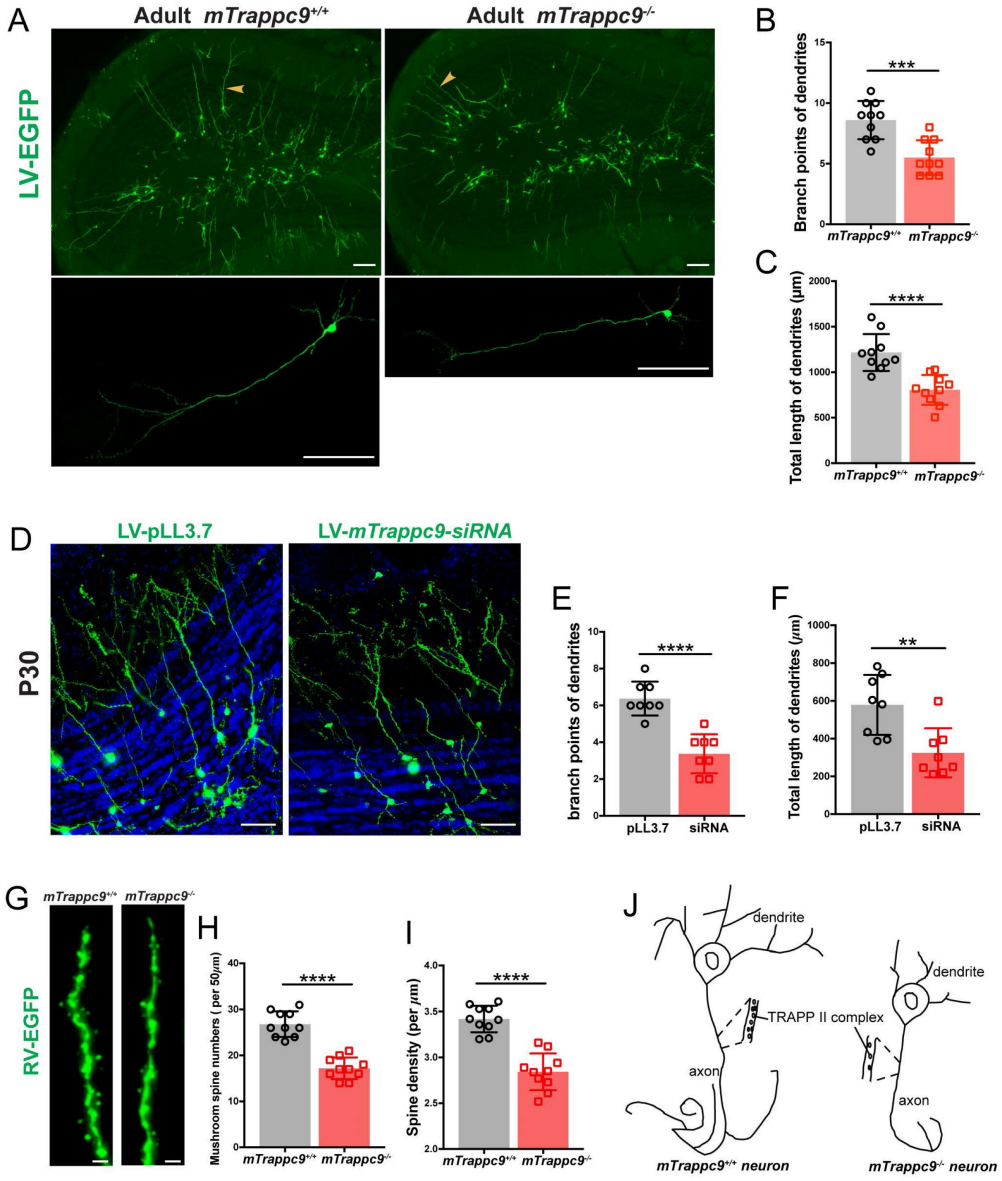
** The length and branches of nerve tracts are reduced after *Trappc9* deficiency *in vivo*. (A-C)**, Lentivirus-EGFP labeled neurons in olfactory bulb of adult* mTrappc9^+/+^* and *mTrappc9^ m/m^* mice. Lower panel shows the amplification result with arrowheads indicating relatively complete neurons. Lentivirus-EGFP was injected into the SVZ of adult mouse brain (2-3 months old), and one month later the EGFP-positive cells were examined. Branch points (**B**) and total lengths (**C**) of dendrites per neuron in the external plexiform layer were quantified. Neurons had smaller branches and shorter lengths in *mTrappc9^m/m^* mice compared to *mTrappc9^+/+^*mice. Representative images from *n*=4 mice/group (**A**); *n*=10 neurons (**B, C**). **(D-F)**, Lentivirus-mediated siRNA knock-down in olfactory neurons in neonatal WT mice. The lentivirus carrying scrambled or *mTrappc9*-targeting siRNA expression cassette was injected intracerebroventricularly in P1 WT mice, and one month later the EGFP-positive cells were examined. The branch points (**E**) and total lengths (**F**) of dendrites in external plexiform layer were reduced in *mTrappc9* knock-down mice. Representative images from n=3 mice/group (**D**); n=8 neurons (**E, F**). **(G-I)**, Retrovirus-EGFP labeled dendrites in hippocampal dentate gyrus of adult *mTrappc9^m/m^* and *mTrappc9^+/+^* mice. (**H**) Mushroom spine numbers of 50 μm terminal ends in labeled neurons; (**I**) the spine density. Dendritic spines were reduced in *mTrappc9^m/m^* mice. Representative images from *n*=3 mice/group (**G**); *n*=10 neurons (**H, I**). (**J**) Graphical illustration depicting the effect of TRAPPII complex on neuronal development. Data are means ± SEM; *t*-test (**B, C, E, F, H, I**); ****P≤0.0001; ***P≤0.001; **P≤0.01; Scale bars: 150 μm (**A**); 50 μm (**D**); 0.5 μm (**G**).

To explore the role of TRAPPII complex in regulating neuritogenesis *in vivo*, we injected EGFP-expressing lentivirus (which labels dividing NSCs/NPCs) into the subventricular zone (SVZ) of adult *mTrappc9^+/+^* and *mTrappc9^m/m^* mouse brains^57^. EGFP-labeled NSCs/NPCs in the SVZ migrate via the rostral migratory stream (RMS) towards the regions of olfactory bulb and therein differentiate into neurons. One month after injections, we analyzed the EGFP-expressing neurons at the matching layer in the olfactory bulb via confocal microscopy. Trappc9 deficient neurons carried shorter dendrites with fewer branches (Figure 7A-C). We performed similar experiments using lentiviral siRNA knockdown system that carries EGFP for cellular tracing^58^. These lentiviral constructs were injected into the right or left ventricle of neonatal mouse brains, where siRNAs can knock down endogenous *mTrappc9* expression in NSCs and NPCs of the SVZ. Compared to the scrambled siRNA control vector-labeled neurons, *mTrappc9* knock-down neurons in the olfactory bulb had dramatically fewer/shorter neurites with fewer branches when examined one month later (Figure 7D-F). The results confirm again that mTrappc9 modulates the neurite elongation and branching of neurons in mouse brains. Considering that the differentiation of *mTrappc9* knock-down neurons undergoes within a wildtype microenvironment constituted by wildtype neural cells, extracellular matrix, and neurites, the data also demonstrates that mTrappc9 modulation intrinsically executes neurite elongation and branching in mature neurons.

To expand this finding, we injected EGFP-expressing retrovirus into the sub-granular zone (SGZ) of the hippocampal dentate gyrus. This technology can measure the development and maturation of adult newborn dentate granule cells (DGCs) incorporating into the existing hippocampal network. The dendritic complexity of EGFP-labeled DGCs was measured by scoring their total dendritic length and their number of branching points. One month after injection, EGFP-expressing DGCs in *mTrappc9^m/m^* mice carried shorter dendrites with fewer branches, similar to our findings in the SVZ (data not shown). We used Neurolucida360 to determine the spine patterns in the dendritic segments of EGFP-labeled granule cells in the outer molecular layer of dentate gyrus. *mTrappc9^m/m^* mouse neurons exhibited a significant reduction in spine density with about 25% fewer spines (Figure 7G, I). The main cortical inputs to the DG arrive from the perforant path axons of neurons in the entorhinal cortex layer, which form synapses onto the DGC dendritic spines, the major postsynaptic site of excitatory synapses. The integration of newborn DGCs relies on dendritic spine growth and maturation. We determined the spine morphogenesis to assess the integration of adult-born DGCs and found that number of mushroom spines, the functional spines, were dramatically decreased in the outer and middle molecular layers in *mTrappc9^m/m^* mice (Figure 7G, H). Taken together, *in vivo* tracing experiments show that *Trappc9* deficiency disrupts TRAPPII complexes, resulting in defective neurite elongating and branching and causing less functional connections between neurons (Figure 7J).

## Discussion

Intellectual disability (ID) is one of the most common developmental disorders, manifesting in up to 3% of the world population before the age of 18^59^. In the past decade, extensive omics studies have shown that genetic and epigenetic factors play key roles in causing these disorders, which are primarily characterized by autosomal dominant (*de novo*) mutations^60^ or by chromosomal aberrations, such as Down's syndrome 61. Recently, genetic screenings in clinical practice have been able to identify disorders associated with non-syndromic autosomal recessive intellectual disability (NS-ARID)^62^-^64^. This includes identifications of new groups of disorders, such as the novel burgeoning category of TRAPPopathies, which are defined by mutations in TRAPP-associated proteins^26^. While genetic screenings help to identify novel genes associated with NS-ARIDs, they cannot always provide the functional consequences of these gene variations for each type of ID, its prognosis, or optimized therapeutical options for families. NIBP syndrome is one type of NS-ARID, caused by autosomal recessive variations and mutations of *TRAPPC9*^24^, leading to a deficiency of functional TRAPPC9.

Patients carrying homozygous or compound heterozygous mutations of *TRAPPC9* genes display distinctive clinical phenotypes mainly including neurological manifestations such as severe intellectual disability predominating on speech, behavioral anomalies, motor development, and variable post-natal microcephaly^29^-^45^. Some patients present with clinical features of autism spectrum disorder^32^, ^33^, ^65^, ^66^. In addition, the characteristic facial appearance of patients is remarkably consistent among NIBP syndrome patients: prominent nasal bridge, full cheeks, short and upturned philtrum, everted lower lip, and prominent and widely spaced upper central incisors. In this study, we use zebrafish and mice as two separate preclinical models to elucidate the mechanisms underlying the neural phenotype of NIBP syndrome. Both models recapitulate the neurodevelopmental phenotypes of patients with NIBP syndrome, in concord with recent reports using *Trappc9* knockout-first^27^, ^34^ or CRISPR/Cas-mediated deletion^46^ mouse models. We focus on embryonic, postnatal, and adult neurogenic mechanisms responsible for microcephaly and white matter reduction after Trappc9 loss of function.

Neurons output and input information via various projections of dendrites and axons. During embryonic and adult neurogenesis, neuronal polarization occurs by the elongation and branching of axons and dendrites, which allows the neurons to establish unique patterns of connectivity in the brain^67^-^70^. This process requires a delicate regulation of extrinsic and intracellular cues that promote and/or inhibit neurite growth, elongation, and branching to guarantee that the appropriate connections are formed with the correct partners necessary for the development of the complex circuits of neurons in the brain. In recent years, significant advances have been made in identifying the extrinsic and intrinsic intracellular factors that control neuronal polarization^71^, ^72^. The intracellular factors involved in neurite elongation and branching include five major classes of proteins: kinases, small GTPases, transcription factors, ubiquitin ligases, and cytoskeleton-associated proteins^73^-^76^. Here we present another intracellular factor, TRAPPII complex, which regulates elongation and branching of both axons and dendrites in neurons. We demonstrate for the first time that disruption of the TRAPPII complex by Trappc9 deficiency results in defects of both axon and dendrite elongation and branching, which may be responsible for the microcephaly and intellectual disability seen in human patients.

The TRAPP complexes^26^, including TRAPPI, TRAPPII, TRAPPIII, are multi-subunit tethering factors, first identified in yeast. TRAPPII is composed of the common subunits Trs20, Bet5, two copies of Bet3, Trs23, Trs31 and Trs33 and four additional subunits (Tca17, Trs65, Trs120 and Trs130). Human TRAPP complexes contain a similar core of proteins (TRAPPC1, TRAPPC2, two copies of TRAPPC3, TRAPPC4, TRAPPC5 and TRAPPC6A/B)^26^. In addition to this core TRAPPI, TRAPPII uniquely adds TRAPPC9 and TRAPPC10. In yeast, TRAPPII is implicated in anterograde traffic through or from the Golgi as well as traffic between endosomes and the Golgi^77^. Additionally, TRAPPII and Ypt31/32 (the homologue of mammalian Rab11) help to tether secretory vesicles or tubulovesicular structures along the cleavage furrow while the exocyst tethers vesicles at the rim of the division plane^78^. Trappc9 knockdown upregulated Rab11 expression but reduced Rab11 activation in one mouse model of mTrappc9 deficiency 46, 79, 80, suggesting that the defects in elongation and branching in Trappc9 deficient animals may result from defects in Rab11 activation. Rab11 recruits Rabin8 through TRAPPII (Trappc9) to the pericentrasomal vesicles and activates Rab8 to promote ciliogenesis^81^-^83^. Thus, we performed single cell RNA sequencing, quantitative proteomics, western blotting, and immunofluorescent staining to detect the expression and distribution of various Rab proteins. However, we did not identify significant alterations of any Rab proteins, even Rab11 (data not shown). This finding was surprising to us as previous data have shown that TRAPPII physically interacts with Rab11, with Trappc9 identified as a major component of this interaction^46^, ^79^, ^80^, ^84^, ^85^. Additionally, ablation of mTrappc9 up-regulated levels of Rab11 in the brains of mTrappc9 KO mice in other m*Trappc9* deficient models^79^. However, since our mouse model, possesses a truncated Trappc9 protein and maintains the TRAPPII complexes in neural cells, this may allow for Rab11 signaling to be unaffected. Further studies are needed to assess the functionality of the truncated Trappc9 protein in our mouse model.

Alternatively, recent reports from other labs have suggested that TRAPPII complexes may be able to form in the absence of Trappc9 if Trappc10 is still present. Since we identified that Trappc10 protein was still detectable in our mTrappc9 deficient mice, it is possible that Trappc10 expression was sufficient to maintain TRAPPII functions. Importantly, recent reports using skin fibroblasts or lymphoblastoid cell lines isolated from patients with TRAPPC9 syndrome, containing a truncation mutation Arg475*, found that there were no observable defects in the early secretory pathway (including Golgi markers such as GM130) and that, while reduced, Trappc10 was still expressed^23^, ^27^. While these studies confirmed the absence of expression of full length TRAPPC9 protein, they did not look at the presence of a truncated form. Additionally, further studies suggested that even at reduced levels, Trappc10 is sufficient to maintain TRAPPII functions, while the opposite is not necessarily true. While one group using a different Trappc9 deficiency mouse model found upregulation of Rab11 expression, they also showed significantly high downregulation of Trappc10, which may have factored into this finding 46, 79, 80.

In the present study, transcriptomics, proteomics, RT-qPCR, western blot analyses, and immunostaining analyses demonstrated that Trappc9 deficiency causes instability of TRAPPII complex in actin filament and microtubules of neurites and growth cones. This finding suggests that Trappc9 plays an essential role in regulating the stability of TRAPPII complex. This is consistent with a recent report that disruption of Trappc10, another unique protein of TRAPPII complex^26^, also induces the instability of TRAPPII complex^27^. Concomitant reduction of Trappc9 and Trappc10 in either deficient model indicates that both regulate each other during the trafficking of TRAPPII complex. While we did find that TRAPPII complex disruption negatively impacted neurite elongation and branching, we did not identify any obvious alteration of Golgi complexes, endosomes, and COPI vesicles by Trappc9 deficiency in neurons of mouse brains. Here, we provide the evidence to show TRAPPII complexes are associated with microtubule/microfilament stability. TRAPPII complexes do not seem to modulate the growth cones that mainly consist of microfilaments. In contrast, newborn neurites and neurite branching are highly dependent on the functions of TRAPPII complexes. As microtubules control the stability of newborn neurites and neurite branching, this suggests that TRAPPII complexes might be involved in the formation and maintenance of microtubules in neurites. While we were unable to work out how TRAPPII complexes modulate the formation and maintenance of microtubules due to their small size, we did observe that microtubules in the tails of sperm cells of *mTrappc9^m/m^* mice were defective (Figure S10A, B). Ultrastructure imaging showed that microtubule-organizing centers were intact, suggesting TRAPPII complexes are involved in the assembly or/and stability of microtubules (Figure S10C). In *Drosophila* dividing spermatocytes, Trappc9 interacts with Rab11 to promote cytokinesis 86-88. Thus, sperm cells may serve as an alternate cellular model that mimics the protein transportation in neurites to address the mechanisms related to how TRAPPII complexes control the elongation and branching of both axons and dendrites during neurodevelopment and maturation.

Potential off-target effects are a well-known concern when using oligonucleotide-mediated knockdown and CRISPR/Cas-based gene editing. To address this, we used multiple approaches to verify that the observed effects on neurite elongation and branching were specific to the knockdown or knockout of *Trappc9*. First, we selected the best scored sgRNA target sites with high efficiency and high specificity using well-established and validated sgRNA design programs such as CRISPick, CHOPCHOP. Morpholino targets were designed by the vendor (Gene Tools) for high specificity and efficiency. We also used multiple independent target sequences targeting different regions of the *Trappc9* gene as well as the scramble sgRNAs as negative control to ensure that the observed effects were not due to off-target effects. Additionally, we observed similar phenotypes using antisense morpholino and CRISR/Cas9 gene editing. In further support of our zebrafish model, we utilized Cre-mediated conditional knockout mouse model, which generally minimizes the likelihood of off-target effects. In many experiments, we used both zebrafish and mice and observed the same phenotype. Finally, we performed transcriptomic analyses to identify potential off-target effects on gene expression and pathways related to neurite outgrowth and did not observe any significant changes in these pathways. In summary, we took several steps to carefully control and exclude the possibility of off-target effects in our study, and we believe that our findings provide compelling evidence for the role of Trappc9 in regulating neurite outgrowth.

In conclusion, Nibp/Trappc9 deficiency impaired the stability of the TRAPPII complexes, resulting in defective elongation (extension) and branching of neuronal dendrites and axons. Trappc9 deficiency did not lead to any deficits of neurite initiation or generation of neural cell number/types in embryonic and adult brains. Our study provides novel genetic/molecular evidence that may help characterize mechanisms underlying the phenotype of patients with a type of non-syndromic autosomal recessive intellectual disability. These animal models can be used to develop therapeutic approaches to treat disorders caused by variations and mutations of *NIBP/TRAPPC9*.

## Methods

### Animals

All experiments involving the use of animals were performed in accordance with National Institutes of Health and approved by the Institutional Animal Care and Use Committee (IACUC) of West China Hospital and Temple University Lewis Katz School of Medicine. Zebrafish and mice were raised and maintained under standard conditions. Briefly, zebrafish were raised on a 14 h light/10 h dark in the water system conditions consisting of pH7.3-7.5, conductivity 500-600 μS, and temperature 28℃. Zebrafish embryos before 4 days post fertilization (dpf) were kept in golf water at 28℃ incubators. Mice were raised in an environment with a temperature of 20℃, humidity 40%-50% and 12 h light/12 h dark cycle.

### Generation of *zTrappc9* Mutant Zebrafish

CRISPR-Cas9 was used to generate *zTrappc9* mutant zebrafish. Suitable target sites were screened by CHOPCHOP (https://chopchop.cbu.uib.no). We constructed 23 targets and finally found two effective target sites (Table S1), located in exon-3 and exon-23 respectively. Synthesized gRNA oligonucleotides were cloned into a pT7-gRNA vector. Subsequently, gRNAs were transcribed *in vitro* and purified for further utilization. A concentration of 250 ng/μL gRNA and 150 ng/μL NLS-Cas9-NLS nuclease were mixed and microinjected into 1-cell stage zebrafish embryos. Mosaic mutant F0 fish was crossbred with AB fish, the progenies (F1) were screened for mutant types. Genotype identification was determined by tail fin clipping, DNA extraction, PCR amplification, agarose gel electrophoresis, cloning and sequencing. We only obtained the fish with mutation at target1, which produced 5 mutants. Genotyping primers for distinguishing homozygous mutant and wild type fish were “Fish 5F” and “Fish 5R” (Table S1).

### Generation of *mTrappc9* Deficient Mice

Traditional recombination technology was used to generate *mTrappc9* conditional knock-out mice. Briefly, a gene targeting vector was made that contained a floxed *mTrappc9* allele in which exon-2 to exon-5 were flanked by two loxP sites and could be deleted by Cre-mediated recombination, resulting in functional deficiency in both isoform I and II of mTrappc9 (Fig. S1G). To construct this vector, we used genomic DNA from G4 ES cells to PCR-amplify 8.6 kb fragments from the *mTrappc9* locus and cloned them into a knock-out vector. The resulting constructs were linearized and electroporated into G4 ES cells, after which G418-resistant ES cells were selected to identify homologous recombination events. Two targeted ES cell lines were identified and expanded. Gene targeted ES cells were injected into B6(Cg)-Tyr^c-2J^/J blastocysts and transferred into the uteri of pseudo-pregnant C57BL/6J female mice. Chimeric males resulting from the injected blastocysts were bred to B6(Cg)-Tyr^c-2J^/J females to generate the targeted floxed *mTrappc9* allele. The targeted allele was further bred to a Rosa26-FLPe line (Jax# 009086) to remove the FRT-flanked Neo cassette. Mice carrying floxed alleles of *mTrappc9* were crossbred with Ella-Cre line (Jax# 003724) to generate *mTrappc9* knock out (*mTrappc9^m/m^*) mice. All *mTrappc9^m/m^* mice used in the experiment were pure background C57BL/6J. Primers for genotyping are: Mouse T5, T8 and T16 (see Table S1).

### Injection of Morpholinos in Zebrafish Embryos

Morpholino antisense oligonucleotides (MO) were used to generate *zTrappc9* knock-down zebrafish. Two splice-blocking MOs were designed (MO1 and MO2) and obtained from Gene Tools (Table S1). MOs were microinjected into 1-cell stage zebrafish embryos with a volume of 1 nL each embryo. The lowest effective concentrations of MO1 and MO2 were 4 ng/μL and 2 ng/μL respectively. Since MO2 was equally effective at a lower concentration, it was selected in the remaining experiments. A pair of primers, “*zTrappc9* RT F” and “*zTrappc9* RT R” was used for qPCR to detect morpholino knock-down efficiency (see Table S1).

### Stereotactic Microinjection of Lentiviral/Retroviral Vectors in Mouse Brain

Three types of microinjections were carried out *in vivo*. We used the 3rd generation packaging systems for lentiviral production including pMDLg/pRRE, CMV-VSV-G and pRSV-Rev as described previously^89^. We used GP2-293 cells (#631458, Takara) for retrovirus preparation of pUX-EGFP vector following the company's protocol. Standard virus concentration, purification and titering were followed. The titer of all the prepared viruses for microinjection was around 1×10^8^ transduction unit (TU)/ml.

Lentivirus-EGFP was used to label NSC/NPC of SVZ in adult *mTrappc9^+/+^* and *mTrappc9^m/m^* mice for the detection of the newborn neurons of olfactory bulb following adult neurogenesis. Adult *mTrappc9^+/+^* and *mTrappc9^m/m^* mice (2-3 months old) were anesthetized with isofluorane and placed in a brain stereotactic instrument (RWD). Stereotactic coordinates for injection were medial-lateral (ML): -1.2 mm; anterior-posterior (AP): +0.7 mm; dorsal-ventral (DV): -2.5 mm from bregma. The microliter syringe was slowly pushed into brain tissue via predrilled holes, and 2 μL lentivirus was slowly injected at a uniform speed. When the injection was complete, the needle was left for two min before slowly withdrawing, and then mice were sutured. After one month of normal feeding, the brains of injected mice were taken for frozen section observation.

Retrovirus-EGFP was used to label the dividing NSC/NPC in the SGZ of dentate gyrus of *mTrappc9^+/+^* and *mTrappc9^m/m^* adult mice (2-3 months old), and the labeled newly born neurons were examined one month later. The stereotactic coordinates were ML: ±1.5 mm; AP: -2.0 mm; DV: -1.9 mm from bregma. The injection procedure was the same as described above.

The lentivirus-pLL3.7-EGFP or lentivirus-*mTrappc9*-siRNA-EGFP was used to label NSC/NPC of SVZ in P1 C57BL/6J mice, and labeled neurons of olfactory bulb were detected after neurogenesis. *mTrappc9*-siRNA and pLL3.7 were used as *mTrappc9* knock-down and control respectively. After placing the pups on ice for 5 min, the lentivirus was injected into lateral ventricles of mice. After one month, animals were perfused for immunohistochemistry and confocal image analysis.

### Primary Cortical Neuron Culture

Neurons from cerebral cortex of E17.5 embryonic mice were directly used for primary neuron culture. Coverslips were coated with poly-L-lysine in 24-well plates. Mouse cerebral cortexes were removed and digested with 0.25% Trypsin-EDTA at 37℃ for 15 min. After digestion, Trypsin-EDTA was removed and plating media (10% FBS, 20% glucose, 1× sodium pyruvate, 1× Glutamax, 1× penicillin/streptomycin in BME media) was added with gentle pipetting for 10 times to facilitate dissociation. Cell suspensions were filtered with a 70 μm cell filter (Falcon) and centrifuged at 1,000×rpm for 5 min. Suspended cells were plated in 24-well plate at a density of 1×10^5^ cells/well in plating media. After 2-4 h of culture, the media was replaced with maintenance media (1× B27, 1× Glutamax, and 1× penicillin/streptomycin in Neurobasal media). Half of the maintenance media was changed every 2-3 days.

### NSC/NPC Culture and Neuronal Differentiation

NSCs/NPCs from cerebral cortex of E13.5 embryonic mice were cultured, and neuronal differentiation was performed as described previously^90^. In brief, the cerebral cortexes were cut into pieces and cell suspensions were centrifuged at 1,000×rpm for 5 min. The dissociated cells were cultured at a density of 2×10^5^ cells/ml in DMEM/F12 proliferation medium containing 20 ng/ml epidermal growth factor (EGF) and 10 ng/ml basic fibroblast growth factor (bFGF) (StemCell Technologies). After appropriate time of culture, primary neurospheres were digested with Accutase and filtered with 70 μm cell filter. The cells were collected and frozen with cryopreservation media (90% proliferation medium and 10% DMSO). When needed for differentiation culture, NSCs/NPCs were recovered and passaged for at least one generation. Coverslips for neuronal differentiation culture were coated with poly-L-lysine and laminin in a 24-well plate beforehand and cells were plated at a density of 1×10^5^ cells/well in proliferation media. After 2 days of culture, the proliferation media was replaced with differentiation media (same as proliferation media, but without EGF and bFGF), and half of the media was changed every day. For cytoskeleton experiments, cells at 6 days of differentiation treated with Latrunculin (50 μM, 2 h), Vinblastine (50 μM, 4 h) or DMSO as the vehicle control in the incubator before immunofluorescence staining.

### Primary Oligodendrocytes Culture

Coverslips for oligodendrocytes culture were coated with poly-L-lysine in a 24-well plate in advance. The cerebral cortexes of P8-P9 mice were removed and digested with Papain. Collected cells were incubated with CD140a antibody and the oligodendrocyte precursor cells (OPC) were sorted by flow cytometry. OPCs were suspended in DMEM-10F media (10% FBS in DMEM media) and were plated at a density of 1-2×10^5^ cells/well. After 2-3 days of culture, the DMEM-10F media were replaced with oligodendrocyte differentiation media (1× Glutamax, 1× sodium pyruvate, 1× SATO, 1× ITS, 1× B27, 0.05 mg/mL *N*-Acetyl-L-cysteine (NAc), 0.1 μg/mL Biotin, 1×cellgo trace element B, 1× penicillin/streptomycin in DMEM and Neurobasal media). Half of the oligodendrocyte differentiation media was changed every 2-3 days.

### Zebrafish Whole Mount *in situ* Hybridization

Probes for whole mount *in situ* hybridization (WISH) were amplified by Q5 DNA polymerase (NEB, M0491L) and cloned into pGEM-T easy vectors. Plasmids were linearized with restriction enzymes (NEB) and transcribed into single-stranded RNA labeled with digoxigenin-11-UTP (Sigma, 11277073910).

Whole experiments were carried out in nuclease-free conditions. Zebrafish embryos were fixed with 4% PFA at 4℃ overnight. Embryos were dehydrated stepwise into methanol and stored at -20℃. When needed for experiments, the embryos were rehydrated stepwise into 1× PBST. Embryos were permeabilized with protease K (10 μg/mL) and washed 3-5 times with 1× PBST. Next, tissues were pre-hybridized with pre-hybridization buffer (2:1:1 ratio of formamide:20×SSC: DEPC:H_2_O with 0.1% Tween-20) at 65℃ for at least 4 h. RNA probes were dissolved in hybridization buffer (10 mg yeast RNA and 1 mg heparin in 20 mL pre-hybridization buffer) at a concentration of 3 ng/μL, then added to embryos and incubated at 65℃ overnight. The next day, the probes were removed, and the embryos were treated stepwise with 50% formamide/2×SSCT, 2×SSCT, 0.2×SSCT, 0.1×SSCT, 0.05×SSCT and MABT buffer. Tissues were incubated in blocking solution (8g blocking reagent in 72mL MAB) at room temperature for 1 h prior to overnight incubation with anti-digoxigenin-AP antibody at 4℃. After removing antibody, the embryos were washed stepwise with 10% serum/MABP, MABT, and detection buffer (100mM Tris-HCl pH 9.5, 50 mM MgCl_2_, 100 mM NaCl, 0.5% Tween-20, 1 mM Levamisole). NBT/BCIP tablets were dissolved in ddH_2_O and added to the embryos for probe labeling. To remove nonspecific labeling, embryos were dehydrated stepwise into methanol at 4℃ overnight, then rehydrated for imaging.

### Immunofluorescence Staining

Four types of samples were used for immunofluorescence: sections on glass slides (12 or 14 μm), floating sections (50 μm), whole mount zebrafish embryos, or cells on coverslip. Adult mice were anesthetized with sodium pentobarbital (45 mg/kg), and perfused transcardially with 1× phosphate buffered saline (PBS) followed by 4% paraformaldehyde (PFA). Brains and/or spinal cords were removed and incubated in 4% PFA at 4℃ overnight. Fixed tissues were dehydrated in 30% sucrose in PBS until the tissues sank to the bottom (24 to 48 h), then embedded in OCT. Sections were cut using a cryostat at a thickness of either 12 or 14 μm and mounted on glass slides or 50 μm and placed in 5-mL tubes. Zebrafish embryos were fixed with 4% PFA at 4℃ overnight. Tissues were embedded with agarose (1.5% agarose and 5% sucrose) and dehydrated with 30% sucrose prior to sectioning. Adherent cells on coverslips were fixed with 4% PFA at room temperature for 20 min.

The procedure for immunofluorescent staining of all four sample types was almost the same, with only minor individual differences. Briefly, samples were washed 3 times with 0.3-0.5% Triton-PBST for 15-20 min each, then incubated in blocking buffer (1% serum and 3% BSA in 0.3% Triton-PBST) for 1 h at room temperature. Samples were then incubated with primary antibodies in blocking buffer at 4℃ overnight, with extended time for the floating sections and whole mount zebrafish embryos. The following day, samples were washed 3 times with PBST for 10 min each, followed by incubation in secondary antibodies in PBST for 1 h at room temperature or 4℃ overnight. Samples were washed 3-5 times with PBST for 10 min each and mounted with Fluoromount-G (Southern Biotech, 0100-01).

For whole mount zebrafish embryos, tissues were penetrated with acetone at -20℃ for 30-40 minute prior to permeabilization. Additionally, after the final washing prior to mounting, zebrafish tissues were incubated stepwise in glycerol for subsequent imaging.

Immunofluorescence samples were imaged with following microscopes: ZEISS Axio Scope A1; ZEISS LSM 900; ZEISS Axio Zoom. V16; NIKON TiA1-N-STROM; and LEICA TCS SP8.

### Co-Immunoprecipitation and Western Blotting

Mouse brain tissues were weighed and homogenized with RIPA buffer (containing protease inhibitor at a ratio of 1:10 (w/v)) through ultrasonication (5-6 times: 20% amplitude, 5s on, 9s off). The lysates were centrifuged at maximum speed in an Eppendorf Centrifuge. The clear lysates were either denatured for 5 min at 98ºC immediately in 1x sample buffer or stored at -80^o^C until use. For co-IP experiment, an equal amount of lysate (500 ug) between groups was incubated with an equal amount of antibodies (TRAPPC3 1:500; TRAPPC9 1:500) at 4 ℃ for 2 h or overnight. Then appropriate equal amount of protein A coated magnetic beads were added and incubate for 1 h at room temperature. After washing with PBS for three times, and the samples were boiled with sample buffer at 98℃ for 10 min. The same volume of denatured protein samples was run in polyacrylamide gels and transferred onto PVDF membranes. Membranes were blocked with blocking buffer (5% non-fat milk in 1× TBST) for 1 h at room temperature and incubated with primary antibodies in 1× TBST 4℃ overnight. The membranes were washed 3 times for 5 min each and incubated with secondary antibodies for 1 h at room temperature. Membranes were washed 3-5 times for 15 min each, then the levels of protein-antibodies were determined with an imaging system (Tanon, 4200R) and analyzed with NIH ImageJ (v1.53) for densitometric measurements. The relative protein levels (ratio) in the immunoprecipitate were normalized by the corresponding protein levels in the input, and the statistic difference in the ratio between *mTrappc9^+/+^* and *mTrappc9^m/m^* mice were analyzed.

### Single-cell RNA-sequencing

Cells for single-cell RNA sequencing were obtained from cerebral cortex of P1 *mTrappc9^+/+^* and *mTrappc9^m/m^* mice born in the same litter. The tissues were digested with Papain Dissociation System according to the manufacturer's protocol. Live cells were sorted using flow cytometry, with a minimum of 2 million cells were collected in each group. Libraries were prepared using BD Rhapsody^TM^ technology. Sequencing platform was Novaseq 6000 PE150 of Illumina. Raw data analysis was performed using R version 4.0.2. as described previously^91^.

### Proteomics

Quantitative proteomics of adult mouse cerebral cortex was performed using TMT-6 labeling technology. The proteins were extracted by liquid nitrogen grinding and sonication. Protein concentrations were determined by BCA protein assay. 50 μg proteins of each sample were taken for trypsin digestion and followed by TMT labeling. Labeled samples were separated on Agilent Zorbax Extend-C18 column using Agilent 1100 HPLC. Mass spectrometry data was analyzed by Proteome Discover 2.4 (Thermo). *mTrappc9^+/+^* and *mTrappc9^m/m^* served as the control and experimental group, respectively, with 3 replicates in each group.

### Reverse Transcription Quantitative PCR (RT-qPCR)

Zebrafish embryos (at least 20 embryos/group) and adult mouse cerebral cortex were collected for RT-qPCR analysis in nuclease-free tubes. For total RNA extraction, TRIzol was used to lyse cells, chloroform was used to separate RNA, and isopropanol was used to precipitate RNA. RNA was reverse transcribed using Primer Script II Reverse. RT-qPCR was performed with FastStart SYBR Green Master (Roche, cat. no. 04673484001) using the CFX96 Real-Time PCR System (Bio-Rad). All primers used for RT-qPCR spanned an exon-exon junction (Table S1). The mRNA expression was quantified relative to *GAPDH* expression.

### Transmission Electron Microscopy

Adult mice were perfused with 2.5% glutaraldehyde in 0.1M phosphate buffer. Brains were fixed with brain mold (RWD) and cut into 1 mm thick slices. Corpus callosum were further dissected out using fine tweezers and fixed with 2.5% glutaraldehyde in 0.1M phosphate buffer at 4℃ for 2-4 h. Tissues progressively underwent post-fixation, dehydration, permeability, embedding, sectioning, and staining. Images were acquired using TECNAI G2 20 TWIN of FEI.

### Mouse Behavioral Assay

Mouse behavioral assay was performed referring to the literature concerned. Behavioral testing started 1 week after onset of treatment. The sequence of the tests was: open field, novel object, fear condition and Barnes maze.

Open field: A 40 cm x 40 cm x 40 cm open-field was utilized in this experiment. The mice were gently dropped in the middle of the open-filed and followed by the video-tracking system for 5 min. The open filed was thoroughly cleaned with 75% alcohol between subjects.

Novel object: Mice were placed in a square arena (40×40×40 cm). A radius of 5 cm around the novel object was defined as the novel object zone. One of the objects was exchanged for a novel object, and the mice explored both old and novel objects for 5 min. After 5 min of free exploration of objects, the mouse was returned to a temporary holding cage. A recognition index (RI) was calculated as the time the mouse spent exploring the new object over total object exploration time. The test arena was carefully cleaned with ethanol between subjects.

Fear conditioning: Fear conditioning were performed using the Animal behavior Conditioning System (Shanghai Jiliang). On the training day, mice were placed into a standard fear-conditioning chamber (lemon, visual cue, and metal chamber floor) for training. Tone- flash-shock pairs were started after an adaptation period of 120 s. Mouse were given a 0.7 mA foot shock for 2s 5s after 30s tone. The tone-flash-shock to mouse was given two times with an interval of 3 min. Following the last tone-flash-shock paired stimulus, mice remained in the chamber for 60 sec before they were removed and placed back to the home cage. To assess memory of context fear conditioning, mice were tested 24 h later in the same conditioning box, in the absence of any foot shock or tone. General activity was assessed by observing activity of mice in the experimental cage for three min, recording the activity and position every 10 sec. Videos of the activity of the mice were recorded for the next 3 mins. After 1 h, the mice were placed into a novel chamber and stayed for 2 min habituation and 30 sec tone stimulus onset.

Barnes maze: The Barnes maze consists of a circular platform 100 cm in diameter raised 100 cm from the ground, with 20 holes (hole diameter: 5 cm) around the maze, and a black metal escape box (19 × 8 × 7 cm) was placed under one of the holes. We place four different shape pictures to help mice memorize the location of escape box around the platform. On Day 0, each mouse was placed at the central of maze and allowed to explore the maze freely for habituation. Mice that did not enter the escape box within 3 min were guided to the correct hole. The mice were trained with four training trials per day for 4 days. Each test trial lasted a maximum of 4 min, with a 15 min break between each trial. During the training, mice were left 10 sec in a dark cylinder (starting chamber) in the center of the maze, allowed to freely explore the maze for a maximum duration of 4 min, and left 1 min in the escape box after each trial. If a mouse failed to enter the escape hole after 240 s, it was guided to the hole and into the escape by an experimenter. During day5 and day12 probe tests, the escape box was removed from the maze, and the mouse was allowed to search freely for 4 min. The analysis of data was completed and recorded by the Smartv3.0 software.

### Quantification and Statistical Analysis

The thickness of cerebral cortex and corpus callosum, the length and branch points of neurites, and cell numbers of mouse cerebral cortex were measured by Fiji. The area of gray/white matter of spinal cord, the inner/outer perimeter of myelinated axons were measured by AutoCAD 2019. Spine number and spine density were measured by Neurolucida 360. Statistical analysis was performed using Graphpad Prism 7, all data are presented as mean ± SEM. Unpaired *t* test, one-way ANOVA and two-way ANOVA were used for statistically significant analysis between two samples in one group, three samples in one group and more than one groups respectively. The threshold of significance was 0.05 (ns, P>0.05; *P<0.05; **P<0.01; ***P<0.001; ****P<0.0001).

## Supplementary Material

Supplementary figures.

## Figures and Tables

**Table 1:** Key Resource Table

REAGENT or RESOURCE	SOURCE	IDENTIFIER
**Antibodies**		
Rabbit anti-SOX2	Abcam	Cat #: ab97959; RRID: AB_2341193
Rabbit anti-TBR2	Abcam	Cat #: ab183991; RRID: AB_2721040
Rabbit anti-LHX2	Abcam	Cat #: ab184337; RRID: AB_2916270
Mouse anti-PSD95	Abcam	Cat #: ab2723; RRID: AB_303248
Rabbit anti-OLIG2	Abcam	Cat #: ab109186; RRID: AB_10861310
Mouse anti-SYT2	Abcam	Cat #: ab154035; RRID: AB_2916272
Chicken anti-SYP	Abcam	Cat #: ab130436; RRID: AB_11156706
Rabbit anti-TRAPPC9	Proteintech	Cat #:16014-1-AP; RRID: AB_2256482
Rabbit anti-TRAPPC3	Proteintech	Cat #:15555-1-AP; RRID: AB_2208142
Rabbit anti-GFAP	Proteintech	Cat #:16825-1-AP; RRID: AB_2109646
Mouse anti-TMEM119	Proteintech	Cat #:66948-1-lg; RRID: AB_2916273
Mouse anti-α-Tubulin	Proteintech	Cat #:66031-1-lg; RRID: AB_2916274
Rabbit anti-TRAPPC10	ABclone	Cat #: A6777; RRID: AB_2767360
Mouse anti-TRAPPC4	Santa Cruz Biotechnology	Cat #: sc-390551; RRID: AB_2916275
Mouse anti-MBP	Santa Cruz Biotechnology	Cat #: sc-71546; RRID: AB_1126436
Mouse anti-RAB5	Santa Cruz Biotechnology	Cat #: sc-46692; RRID: AB_628191
Mouse anti-Acetylated α-Tubulin	Santa Cruz Biotechnology	Cat #: sc-23950; RRID: AB_628409
Chicken anti-NFH	Aves Labs	Cat #: Nfh877982; RRID: AB_2916276
Chicken anti-MAP2	Aves Labs	Cat #: AB_2313549; RRID: AB_2916296
Chicken anti-PAX6	Developmental Studies Hybridoma Bank	Cat #: AB 528427; RRID: AB_2916279
Mouse anti-SYN	Developmental Studies Hybridoma Bank	Cat #: AB_528479; RRID: AB_2916280
Mouse anti-NeuN	Millipore Sigma	Cat #: MAB377; RRID: AB_2298772
Anti-Digoxigenin-AP, Fab fragments	Millipore Sigma	Cat #: 11093274910; RRID: AB_2734716
Mouse anti-GAPDH	Beyotime	Cat #: AF0006; RRID: AB_2715590
Rabbit anti-GFP	Chromotek	Cat #: PABG1; RRID: AB_2749857
Mouse anti-GM130	BD Bioscience	Cat #: 610822; RRID: AB_398141
Rat anti-BCL11b	Biolegend	Cat #: 650601; RRID: AB_10896795
APC anti-mouse CD140a	Biolegend	Cat #: 135907; RRID: AB_2043969
Phalloidin-Fluorescent 555	Cytoskeleton	Cat #: PHDH1-A; RRID: AB_2916284
Donkey anti-mouse Alexa488	Thermo Fisher Scientific	Cat #: A-21202; RRID: AB_141607
Donkey anti-mouse Alexa555	Thermo Fisher Scientific	Cat #: A-31570; RRID: AB_2536180
Donkey anti-rabbit Alexa488	Thermo Fisher Scientific	Cat #: A-21206; RRID: AB_2535792
Goat anti-rabbit Alexa555	Thermo Fisher Scientific	Cat #: A-21428; RRID: AB_2535849
Goat anti-rat Alexa555	Thermo Fisher Scientific	Cat #: A-21434; RRID: AB_2535855
Goat anti-Chicken Alexa 488	Thermo Fisher Scientific	Cat #: A-32931; RRID: AB_2662843
**Experimental Models: Organisms/Strains**		
Zebrafish: AB wild-type	China zebrafish resource center	N/A
Zebrafish:* zTrappc9^m/m^*	This study	N/A
Mouse: Rosa26-FLPeR	The Jackson Laboratory	Strain #: 016226
Mouse: B6(Cg)-Tyr^c-2J^/J	The Jackson Laboratory	Strain #: 000058
Mouse: C57BL/6J	The Jackson Laboratory	Strain #: 000664
Mouse: Ella-Cre	The Jackson Laboratory	Strain #: 003724
Mouse: *mTrappc9^m/m^*	This study	N/A
**Oligonucleotides**		
Morpholinos, gRNA targets, primers sequence provided in Table S1	This study	N/A
pLenti-hU6-EGFP	GENE	N/A
pLenti-pLL3.7-GFP	(Hu et al., 2005)^20^	N/A
pLenti-*mTrappc9*-siRNA-GFP	(Hu et al., 2005)^20^	N/A
pRetro-GFP-puro	GENE	N/A
pMD 18-T simple	Takara	Cat #: D103A
pGEM-T easy	Promega	Cat #: A1360
**Critical commercial assays**		
NucleoSpin Gel and PCR Clean-up Kit	Macherey-Nagel	Cat #: 740609.250
NucleoSpin RNA, Mini Kit	Macherey-Nagel	Cat #: 740955.50
PrimerScript II Reverse Transcriptase	Takara	Cat #: 2690A
Riboprobe^®^ System	Promega	Cat #: P1420/P1440
FastStart DNA Master SYBR Green	Roche	Cat #: 12239264001
**Chemicals, peptides, and recombinant proteins**		
Cas9 nuclease	GenScript	Cat #: Z03469
DAPI	Thermo Fisher Scientific	Cat #: 62248
Fetal Bovine Serum (FBS)	Gemini Bio	Cat #: 900-108
Sodium Pyruvate	Gibco	Cat #: 11360070
GlutaMAX	Gibco	Cat #: 35050061
Penicillin/Streptomycin	HyClone	Cat #: SV30010
BME media	Gibco	Cat #: 21010046
Neurobasal media	Gibco	Cat #: 21103049
DMEM/F-12 media	Cytiva	Cat #: SH30023.01
DMEM media	Gibco	Cat #: 11960044
FGF	Gibco	Cat #: PHG0024
EGF	Gibco	Cat #: PHG0311
DMSO	MP	Cat #: 02196055-CF
ITS	Gibco	Cat #: 41400045
*N*-Acetyl-L-cysteine (NAc)	Millipore Sigma	Cat #: A7250
Biotin	Millipore Sigma	Cat #: B4639
cellgo trace element B	CORNING	Cat #: 25-022-C1
B-27^TM^ Supplement	Gibco	Cat #: 17504044
StemPro^TM^ Neural Supplement	Gibco	Cat #: A1050801
Protease K	Gold Biotechnology	Cat #: P-480-100
Papain Dissociation System	Worthington Biochemical	Cat #: LK003178
**Deposited data**		
scRNAseq raw data	https://ngdc.cncb.ac.cn/search/?dbId=gsa&q=+CRA007035	GSA ID: CRA007035
Proteomics raw data	https://ngdc.cncb.ac.cn/search/?dbId=&q=OMIX001211	OMIX ID: OMIX001211
**Software and algorithms**		
Fiji	National Institute of Health	Version: 2.3.0/1.53q
GraphPad Prism	GraphPad Software	Version: 7.0a
Primer Premier	Premier Biosoft	Version: 5
SnapGene Viewer	SnapGene	Version: 4.3.4

## Abbreviations

COPI: coat protein I
DGC: dentate granule cell
GFAP: Glial fibrillary acidic protein
ID: intellectual disability
Map2: microtubule-associated protein 2
MBP: myelin basic protein
MN: motor neuron
MO: morpholino
NFH: neurofilament heavy chain
NIBP: NIK and IKK2 binding protein
NPC: neural progenitor cell
NS-ARID: non-syndromic autosomal recessive intellectual disability, NSC, neural stem cell
olig2: oligodendrocyte transcription factor 2
OPC: oligodendrocyte precursor cell
PLP: myelin proteolipid protein
PSD-95: postsynaptic density protein 95
RMS: rostral migratory stream
scRNA-seq: single-cell RNA sequencing
SGZ: subgranular zone
SVZ: subventricular zone
TRAPP: transport protein particles
WISH: whole mount *in situ* hybridization
