# Supplementary material for: Defective neurite elongation and branching in Nibp/Trappc9 deficient zebrafish and mice
Source: Int J Biol Sci. 2023 Jun 19;19(10):3226–48. doi: 10.7150/ijbs.78489 (PMC10321293; doi:10.7150/ijbs.78489)
Supplement: Supplementary file 1 — Supplementary figures. [file ijbsv19p3226s1.pdf]

Figure S1. Generation of *Trappc9* deficient animal models.

(A), Schematic illustration of two *zTrappc9* morpholino knock-down constructs. MO1 targets within exon 2, and MO2 targets between exon 3 and subsequent introns. Both morpholinos modify mRNA splicing.

(B-C), RT-qPCR analysis of the knock-down efficiency for two morpholinos in 24 hpf zebrafish (**B**) and time course of MO2 (**C**). *n*=3 experiments.

(D), Schematic illustration of CRISPR/Cas9-mediated *zTrappc9* gene editing. There were 5 mutants identified in Target 1 (located within exon 3), with mutant 5 leading to the most significant change in *zTrappc9* mRNA sequence, labeled as $zTrappc9^{m/m}$.

(E), Sequencing results of the five different mutants after testing in zebrafish.

(F), The majority of mutant F3 eggs did not divide and remained in the 1-cell stage. *n*=20 pairs of F2 zebrafish, and 19 pairs of AB zebrafish.

(G), Schematic illustration of Cre-loxP-mediated *mTrappc9* conditional knock-out (KO) at exon 2 to exon 5, resulting in functional deficiency of isoform II with initial ATG at exon 2 (1139 aa, predicted molecular weight 125.4 kDa) and isoform I with initial ATG at exon 3 (960 aa, 106 kDa), while the downstream transcript may be translated into a truncated protein (787 aa, 87.4 kDa) due to the alternative ATG site at exon 7.

(H), Identification of *mTrappc9* deletion by PCR genotyping using two different primer pairs. The presence of a band via PCR byT5/T8 (intron 1 to exon 2) indicates a wildtype ($mTrappc9^{+/+}$) allele; the presence of a band via PCR by T5/T16 (intron 1 to intron 5, downstream of the deletion site) indicates a knockout (KO, $mTrappc9^{m/m}$) allele. If both bands are present, this indicates a heterozygous genotype ($mTrappc9^{+/m}$).

(I), Body weight increase in $mTrappc9^{m/m}$ mice as compared with $mTrappc9^{+/+}$ mice starting at 7 weeks old. *n*=6 mice/group.

(J), Absence of full-length mTrappc9 protein in the brain of $mTrappc9^{m/m}$ mice detected by western blot analysis. *n*=3 mice/group.

(K), Co-immunoprecipitation and western blot analysis with a mTrappc9 C-terminal antibody, which showed the absence of full-length mTrappc9 protein (~130 kDa), but very minor expression of a truncated mTrappc9 (~90 kDa) in $mTrappc9^{m/m}$ mice. Meanwhile, mTrappc9 can pull down mTrappc10 in $mTrappc9^{m/m}$ mice, indicating that the truncated mTrappc9 has potential for assembling a TRAPPII-like complex. *n*=10 experiments

(L), RT-qPCR analysis of mRNA expression between exons 2-5 (KO) and after exon 6 (new start primer at exon 13-15). The results showed that there was mRNA expression in the C-terminal sequence of mTrappc9. *n*=3 experiments.

Data are means ± SEM; *t*-tests (**F**); One-way ANOVA (**B**); Two-way ANOVA (**C, I, L**); ****P≤0.0001; **P≤0.01; ns, not significant (P>0.05).

Figure S2. Phenotypes of zebrafish and mice after Trappc9 deficiency.

(A-B), The embryonic brain sizes of *zTrappc9* mutant and morphant zebrafish were smaller than AB (wildtype) zebrafish. Representative images from *n*=60-200 embryos/group in 11 experiments (**A**); *n*=10 embryos (**B**).

(C-D), The adult brain size of *zTrappc9* mutant zebrafish was significantly smaller than AB (wildtype) zebrafish. Representative images from *n*=3 zebrafish/group.

(E-H), There was no significant difference in the brain size of P1 $mTrappc9^{m/m}$ mice (**E, F**),

but a significant decrease in the brain size of P20 *mTrappc9*$^{m/m}$ mice was observed as compared with corresponding littermates (**G, H**). Representative images from *n*=3 mice/group.

(I-L), The brain and spinal cord (thoracic) of adult *mTrappc9*$^{m/m}$ mice were significantly smaller than WT (C57BL/6 wildtype) and *mTrappc9*$^{+/+}$ mice. Representative images from *n*=3 mice/group (**I, J**), *n*=10 mice/group (**K, L**).

Data are means ± SEM; *t*-tests (**D, F, H, J, L**); Two-way ANOVA (**B**); ****P≤0.0001; **P≤0.01; ns, not significant (P>0.05). Scale bars: 100 μm (**A**); 1 mm (**C**); 2 mm (**E, G, I, K**).

Figure S3. Behavioral tests of adult *mTrappc9*$^{+/+}$ and *mTrappc9*$^{m/m}$ mice.

(A-B), Open field test. Both the total moving distance (**A**) and the average moving speed (**B**) of *mTrappc9*$^{m/m}$ mice were reduced compared to *mTrappc9*$^{+/+}$ mice, indicating reduced spontaneous exploration activity of *mTrappc9*$^{m/m}$ mice. *n*=10 mice/group.

(C), Novel object recognition test. *mTrappc9*$^{m/m}$ mice spent less time exploring novel objects compared to *mTrappc9*$^{+/+}$ mice. *n*=10 mice/group.

(D-E), Barnes maze test. The escape latency across 4 training sessions on the first day of experiments was significantly longer in *mTrappc9*$^{m/m}$ mice than *mTrappc9*$^{+/+}$ mice, indicating decreased learning ability (**D**). In the probe test, the short-term memory of *mTrappc9*$^{m/m}$ mice was not affected, but the long-term memory ability decreased significantly as compared to *mTrappc9*$^{+/+}$ mice (**E**). *n*=11-12 mice/group.

(F), Contextual fear conditioning test. Freezing time is decreased in *mTrappc9*$^{m/m}$ mice, indicating decreased contextual fear response and impaired learning. *n*= 10 mice/group.

Data are means ± SEM; t-tests (**A, B, C, F**); Two-way ANOVA (**D, E**); ****P≤0.0001; ***P≤0.001; *P≤0.05; ns, not significant (P>0.05).

Figure S4. The number and type of neural cells in the brains of *Trappc9* deficient zebrafish and mice do not show significant change.

(A-B), RNA *in situ* hybridization and RT-qPCR analysis of *sox2* mRNA expression in zebrafish embryos identified no significant difference among the three groups. Representative images from *n*=20-26 embryos/group in 10 experiments (**A**); *n*=3 experiments (**B**).

(C-E), SOX2 immunostaining of E13.5 mouse cerebral cortex failed to detect any significant difference in SOX2^{+} cell number (**D**) or thickness of ventricular and subventricular layers in *mTrappc9*$^{m/m}$ mice (**E**). Representative images from *n*=3 mice/group (**C**); *n*=5-8 sections (**D, E**).

(F-H), PAX6 and TBR2 immunostaining of E15.5 mouse cerebral cortex. PAX6 labels NSCs of the subventricular zone (SVZ), and TBR2 labels NPCs. Cell number (**G**) and thickness (**H**) of PAX6^{+}/TBR2^{+} cell layers did not show significant difference in *mTrappc9*$^{m/m}$ mice. Representative images from *n*=3 mice/group (**F**); *n*=4-6 sections (**G, H**).

(I-L), NeuN (neurons) and OLIG2 (oligodendrocytes) immunostaining of adult mouse cerebral cortex and thoracic spinal cord (**K**)/(**L**) and corresponding cell number statistics of (**I**)/(**J**). The number of neurons and oligodendrocytes did not show significant difference in *mTrappc9*$^{m/m}$ mice compared to control groups. Representative images from *n*=3 mice/group (**I, J**); *n*=7 sections (**K**); *n*=5 sections (**L**).

(M, N), Western blot analysis of adult mouse cerebral cortex, and integrated density of protein bands ratio to GAPDH (**N**). There was no significant difference in the protein expression of

GFAP, NeuN, OLIG2 (oligodendrocytes), or TMEM119 (microglia) in *mTrappc9*$^{m/m}$ mice. *n*=3 mice/group.

(O-R), GFAP immunostaining of adult mouse corpus callosum and thoracic spinal cord to visualize astrocytes (**Q**)/(**R**) and the corresponding area ratio of GFAP fluorescence (**O**)/(**S**). No significant difference was detected among the three groups. Representative images from *n*=3 mice/group (**O, P**); *n*=10 sections (**Q**); *n*=5 sections (**R**).

(S-V), TMEM119 immunostaining of adult mouse corpus callosum and thoracic spinal cord to visualize microglia (**U**)/(**V**) and the corresponding area ratio of TMEM119 fluorescence (**S**)/(**T**). No significant difference was detected among the three groups. Representative images from *n*=3 mice/group (**S, T**); *n*=18 sections (**U**); *n*=5 sections (**V**).

Data are means ± SEM; *t*-tests (**D, E**); One-way ANOVA (**Q, R, U, V**); Two-way ANOVA (**B, G, H, K, L, N**); ns, not significant (P>0.05). Scale bars: 25 μm **(F);** 50 μm **(A, C, O, S**); 150 μm **(I, J, P, T**).

Figure S5. Synapses are reduced after *Trappc9* knock-out.

(A, B), SYN labeled pan-synapse of adult mouse cerebral cortex **(A)** and corresponding area percentage of SYN fluorescence **(B)**. The area of SYN fluorescence is reduced in *mTrappc9*$^{m/m}$ mice, indicating reduction of synapses. Representative images from *n*=3 mice/group (**A**); *n*=8 sections (**B**).

(C, D), SYP labeled pre-synapse of adult mouse cerebral cortex **(C)** and corresponding quantification of SYP$^+$ puncta per area **(D)**. The number of puncta is reduced in *mTrappc9*$^{m/m}$ mice, indicating fewer number of synapses. Representative images from *n*=3 mice/group (**C**); *n*=10 sections (**D**).

(E, F), PSD95 labeled post-synapse of adult mouse cerebral cortex **(E)** and corresponding area percentage of PSD95 fluorescence **(F)**. The area of PSD-95 fluorescence is reduced in *mTrappc9*$^{m/m}$ mice, indicating reduced expression of excitatory post-synaptic receptors that may correspond to a decrease in the number of synapses. Representative images from *n*=3 mice/group (**E**); *n*=8 sections (**F**).

(G, H), SYT2 labeled post-synapse of adult mouse cerebral cortex **(G)** and corresponding area percentage of SYT2 fluorescence **(H)**. The area of SYT2 fluorescence is reduced in *mTrappc9*$^{m/m}$ mice, indicating reduced expression of post-synaptic receptors that may correspond to a decrease in the number of synapses. Representative images from *n*=3 mice/group (**G**); *n*=9 sections (**H**).

Data are means ± SEM; One-way ANOVA (**B, D, F, H**); ****P≤0.0001; *P≤0.05. Scale bars: 20 μm (**C**); 50 μm (**A, E**); 150 μm (**G**).

Figure S6. Altered expression of genes and proteins related to neurites projection in *Trappc9* deficient zebrafish and mice.

(A, B), Single cell RNA sequencing of P1 mouse cerebral cortex. (**A**) t-SNE map of *mTrappc9*$^{+/+}$ (*n*=34407 cells) and *mTrappc9*$^{m/m}$ (*n*=34504 cells). Single cells are colored based on different neural cell types. (**B**) Proportion of each cluster in two groups. There was no significant difference in the proportion of neural cell types between *mTrappc9*$^{+/+}$ and *mTrappc9*$^{m/m}$ mice.

(C, D), Significantly enriched pathways of single cell RNA sequencing. Many of the down-

regulated pathways (**C**) and up-regulated pathways (**D**) were related to axonal and synaptic functions.

(E), RT-qPCR analysis of NF-κB downstream genes in 24 hpf zebrafish embryos. Only 3 genes had statistically significant changes and may contribute to neurite hypoplasia. *n*=3 experiments.

(F), Volcano map of differential proteins detected by proteomics: blue and red dots represent downregulation and upregulation, respectively. Many TRAPPII complex subunits were significantly downregulated. *n*=3 mice/group.

(G, H), Significantly enriched pathways of quantitative proteomics, displaying down-regulated **(G)** and up-regulated **(H)** pathways. Many pathways were related to axon development, synapses, and vesicles.

Data are means ± SEM; Two-way ANOVA (**E**); *P≤0.05; ns, not significant (P>0.05).

Figure S7. The correlation of TRAPPII components with Golgi apparatus, COPI, and endosomes is not affected by *Trappc9* deficiency in neuronal cultures.

(A, B), Trappc9/Trappc10 and GM130 (*cis*-Golgi) immunostaining of NSC-differentiated neurons **(A)** and corresponding area of GM130 positivity in the cell body **(B)**. *n*=4 coverslips/group (**A**); *n*=13 neurons (**B**).

(C, D), Trappc9/Trappc10 and COPG (γ-COPI) immunostaining of primary cultured neurons **(C)** and corresponding expression area of COPI in the cell body (**D**). *n*=5 coverslips/group (**C**); *n*=15 neurons (**D**).

(E-I), Trappc9/Trappc10 and RAB5 (early endosomes) immunostaining of NSC-differentiated neurons. Due to the spatial overlap, it's difficult to evaluate the co-localization between Trappc9/Trappc10 and RAB5 by simple quantification. Instead, we used a co-localization coefficient between the two fluorescent channels, with the results showing that there were no significant differences between *mTrappc9*$^{+/+}$ and *mTrappc9*$^{m/m}$ neurons. *n*=7 coverslips/group (**E**); *n*=16 growth cones (**F-I**).

Data are means ± SEM; Two-way ANOVA (**B, D, F, G, H**); ns, not significant (P>0.05); Scale bars: 10 μm (**A, C, E**).

Figure S8. The distribution of Trappc9 is reduced in *Trappc9* deficient neurons.

(A, B), Trappc9/Trappc10 immunostaining of neurons differentiated from NSCs of E13.5 mouse cerebral cortex cultured for 6 days. While there was a decrease in mTrappc9 in the neuronal cell body, there was no significant changes in the intensity of mTrappc10. Representative images from *n*=8 coverslips/group (**A**); *n*=22 neurons (**B**).

(C-F), Trappc9 and F-actin/α-tubulin immunostaining of mouse NSC-differentiated neurons cultured for 6 days showing the number of mTrappc9 puncta per micron of nascent neurites decreased significantly. Arrowheads indicated several newborn neurites, which showed the reduction in the distribution of Trappc9 in *mTrappc9*$^{m/m}$ neurons compared to *mTrappc9*$^{+/+}$. Representative images from *n*=8 coverslips/group (**C, E**); *n*=33 nascent neurites (**D, F**).

Data are means ± SEM; *t*-tests (**B, D, F**); ****P≤0.0001; Scale bars: 10 μm (**A, C, E**).

Figure S9. The puncta distribution of Trappc9 and Trappc10 along microfilaments/microtubules is reduced in neurons treated with Latrunculin and

Vinblastine.

(A), Latrunculin and vinblastine treated *mTrappc9*$^{+/+}$ neurons labeled by F-actin and α-tubulin. NSC-differentiated neurons at day 6 were treated with latrunculin (5 μM, 2h) or vinblastine (5 μM, 4h). Latrunculin and Vinblastine treatments impair the growth of microfilaments and microtubules respectively. Representative images from *n*=3 coverslips/group.

(B, D), Trappc10 and F-actin (microfilaments) immunostaining. The number of Trappc10^{+} puncta along microfilaments was reduced in neurons after latrunculin treatment. Representative images from *n*=3 coverslips/group (**B**); *n*=18 neurons (**D**).

(C, E), Trappc10 and α-tubulin (microtubules) immunostaining. The number of Trappc10^{+} puncta along microtubules was reduced in neurons after vinblastine treatment. Representative images from *n*=3 coverslips/group (**C**); *n*=18 neurons (**E**).

(F, G), Trappc9 and F-actin immunostaining. The number of Trappc9^{+} puncta distributed along the microfilament in neurons was decreased after treatment with latrunculin. Representative images from *n*=3 coverslips/group (**F**); *n*=18 neurons (**G**).

(H, I), Trappc9 and α-tubulin immunostaining. The number of Trappc9^{+} puncta distributed along the microtubules in neurons was decreased after treatment with Vinblastine. Representative images from *n*=3 coverslips/group (**H**); *n*=18 neurons (**I**).

Data are means ± SEM; *t*-tests (**D, E, G, I**); ****P≤0.0001; Scale bars: 10 μm (**A, B, C, F, H**).

Figure S10. Sperm cell development is inhibited in *mTrappc9*$^{m/m}$ mice.

(A, B), Immunostaining of α-tubulin to label the tail of sperm cells in testicular sections of mice at 20 weeks old, showing a reduction in sperm cell number in *mTrappc9*$^{m/m}$ mice compared to *mTrappc9*$^{+/+}$ mice. Representative images from *n*=3 mice/group (**A**); *n*=7 sections (**B**).

(C), Epididymis section of mice at 10 weeks old. The red arrowheads point out the cross section of several sperm tails showing the normal "9+2" structure in the tail of *mTrappc9*$^{m/m}$ sperm as compared to tail of *mTrappc9*$^{+/+}$ sperm. Representative images from *n*=3 mice/group.

Data are means ± SEM; *t*-tests (**B**); ****P≤0.0001; Scale bars: 50 μm (**A**); 500 nm (**C**).

Figure S1

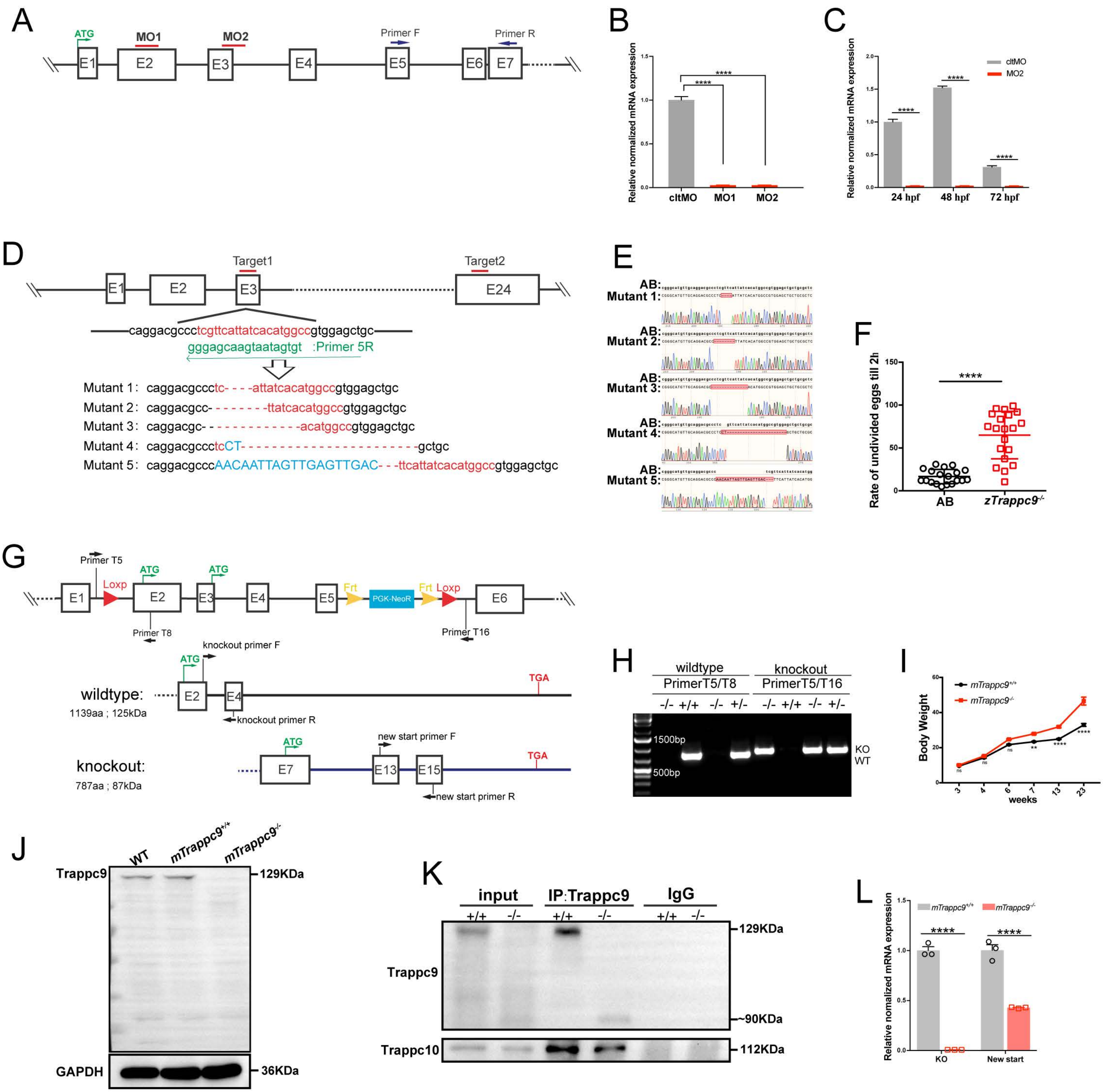

A
ATG
MO1
MO2
Primer F
Primer R
E1 E2 E3 E4 E5 E6 E7
B
Relative normalized mRNA expression
cltMO MO1 MO2
C
cltMO
MO2
24 hpf 48 hpf 72 hpf
D
Target1
Target2
E1 E2 E3 E24
caggacgccctcgttcattatcacatggccgtggagctgc
gggagcaagtaatagtgt :Primer 5R
Mutant 1: caggacgccctc- - -attatcacatggccgtggagctgc
Mutant 2: caggacgcc- - - - - - - -ttatcacatggccgtggagctgc
Mutant 3: caggacgc- - - - - - - - - - - - -acatggccgtggagctgc
Mutant 4: caggacgccctcCT- -gctgc
Mutant 5: caggacgcccAACAATTAGTTGAGTTGAC- - -ttcattatcacatggccgtggagctgc
E
AB:
Mutant 1:
Mutant 2:
Mutant 3:
Mutant 4:
Mutant 5:
F
Rate of undivided eggs till 2h
AB
zTrappc9-/-
G
Primer T5
Loxp
ATG
E1 E2 E3 E4 E5 E6
Frt
PGK-NeoR
Frt Loxp
Primer T8
Primer T16
knockout primer F
wildtype:
1139aa ; 125kDa
TGA
knockout primer R
knockout:
787aa ; 87kDa
E7 E13 E15
new start primer F
new start primer R
H
wildtype
PrimerT5/T8
knockout
PrimerT5/T16
-/- +/+ -/- +/- -/- +/+ -/- +/-
1500bp
500bp
KO
WT
I
mTrappc9+/+
mTrappc9-/-
Body Weight
weeks
J
WT mTrappc9+/+ mTrappc9-/-
Trappc9
129KDa
GAPDH
36KDa
K
input
IP:Trappc9
IgG
+/+ -/- +/+ -/- +/+ -/-
Trappc9
129KDa
~90KDa
Trappc10
112KDa
L
mTrappc9+/+
mTrappc9-/-
Relative normalized mRNA expression
KO
New start

Figure S2

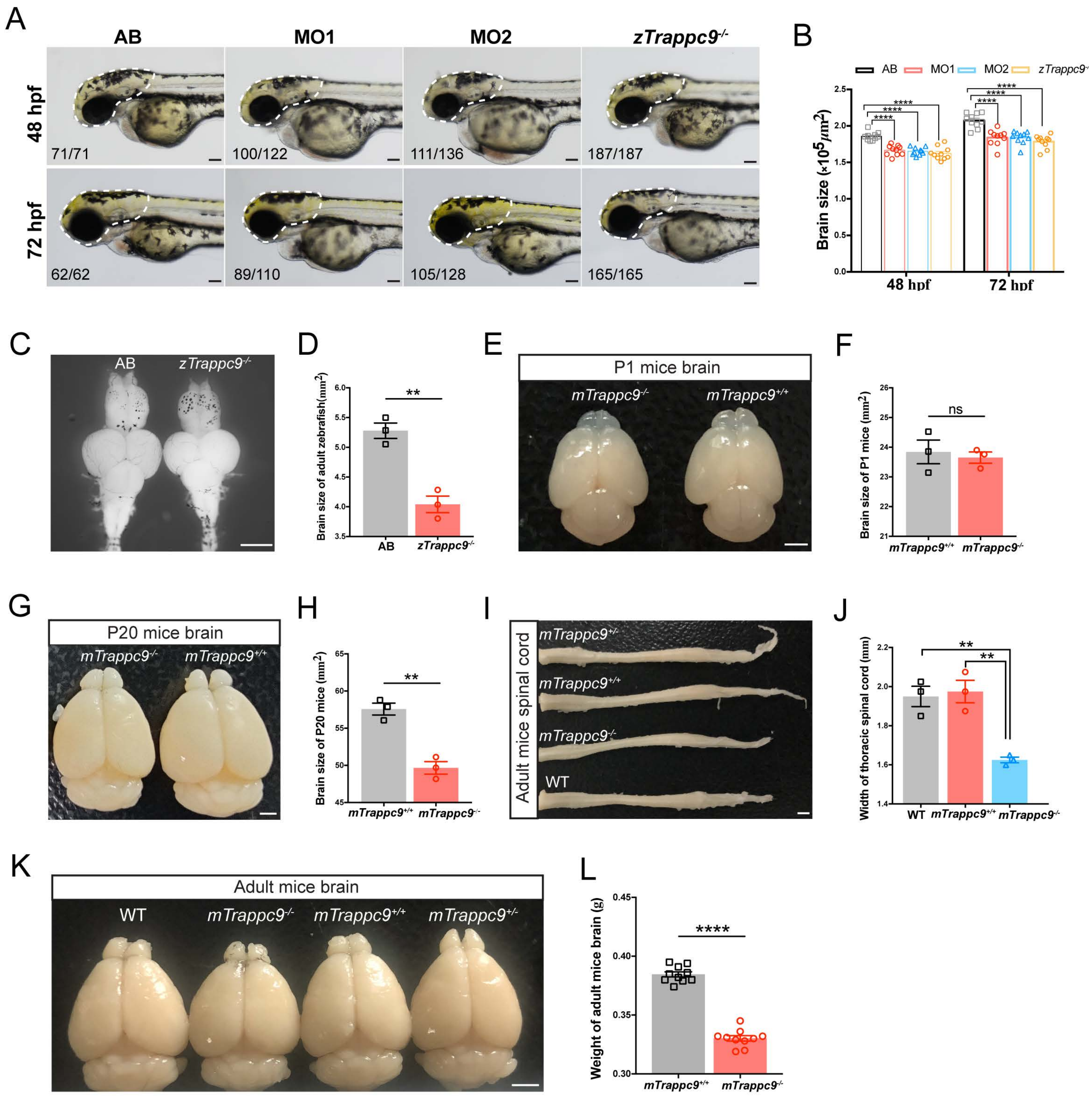
A
AB
MO1
MO2
zTrappc9-/-
48 hpf
72 hpf
71/71
100/122
111/136
187/187
62/62
89/110
105/128
165/165
B
AB
MO1
MO2
zTrappc9-/-
Brain size (×10^5 μm^2)
2.5
2.0
1.5
1.0
0.5
0.0
****
48 hpf
72 hpf
C
AB
zTrappc9-/-
D
Brain size of adult zebrafish(mm^2)
6.0
5.5
5.0
4.5
4.0
3.5
**
AB
zTrappc9-/-
E
P1 mice brain
mTrappc9-/-
mTrappc9+/+
F
Brain size of P1 mice (mm^2)
26
25
24
23
22
21
ns
mTrappc9+/+
mTrappc9-/-
G
P20 mice brain
mTrappc9-/-
mTrappc9+/+
H
Brain size of P20 mice (mm^2)
65
60
55
50
45
**
mTrappc9+/+
mTrappc9-/-
I
Adult mice spinal cord
mTrappc9+/-
mTrappc9+/+
mTrappc9-/-
WT
J
Width of thoracic spinal cord (mm)
2.2
2.0
1.8
1.6
1.4
**
**
WT
mTrappc9+/+
mTrappc9-/-
K
Adult mice brain
WT
mTrappc9-/-
mTrappc9+/+
mTrappc9+/-
L
Weight of adult mice brain (g)
0.45
0.40
0.35
0.30
****
mTrappc9+/+
mTrappc9-/-

Figure S3

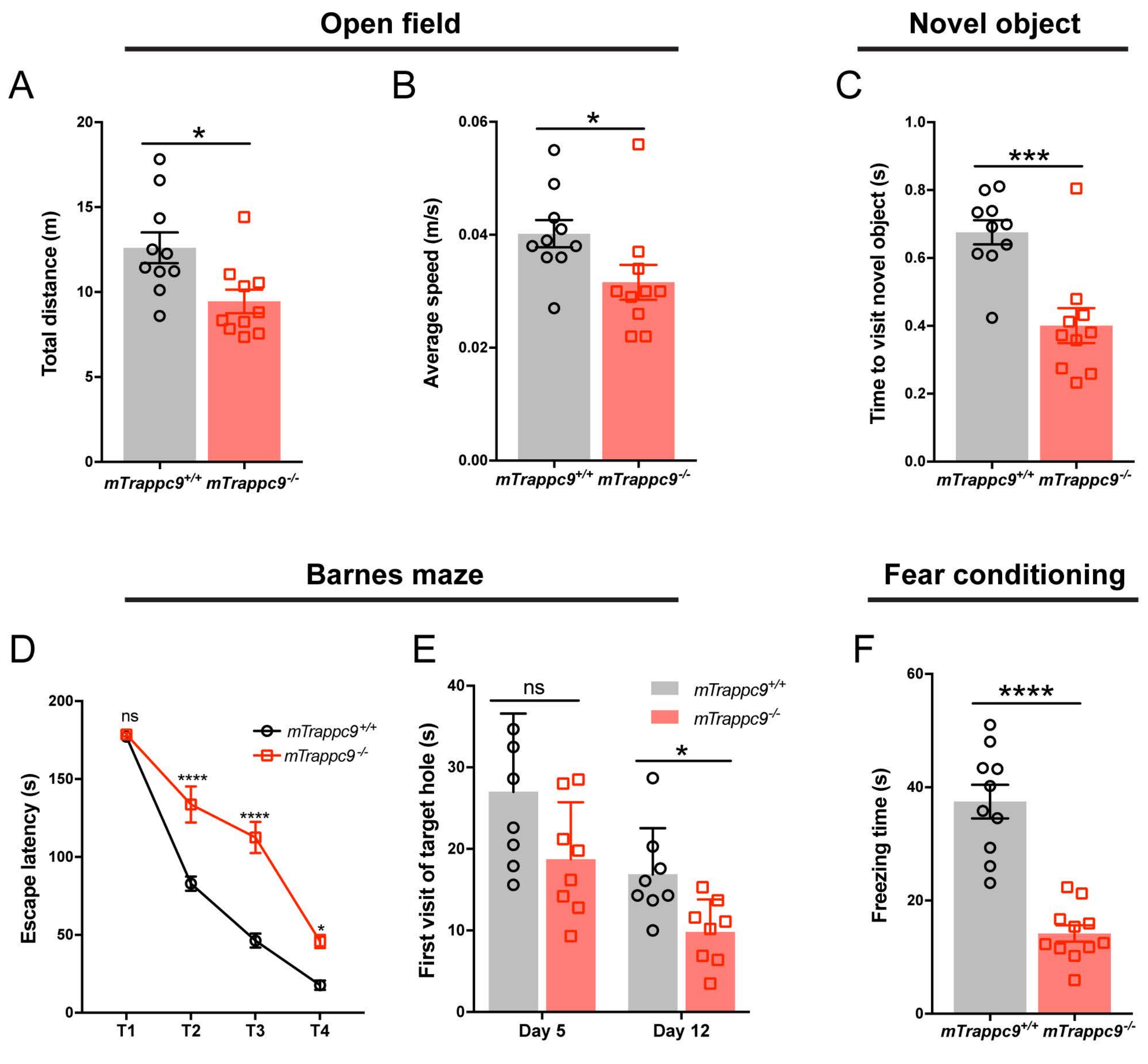
Open field
Novel object
A
B
C
Total distance (m)
Average speed (m/s)
Time to visit novel object (s)
*
*
***
mTrappc9+/+
mTrappc9-/-
Barnes maze
Fear conditioning
D
E
F
Escape latency (s)
ns
****
****
*
T1
T2
T3
T4
First visit of target hole (s)
ns
*
Day 5
Day 12
Freezing time (s)
****

Figure S4

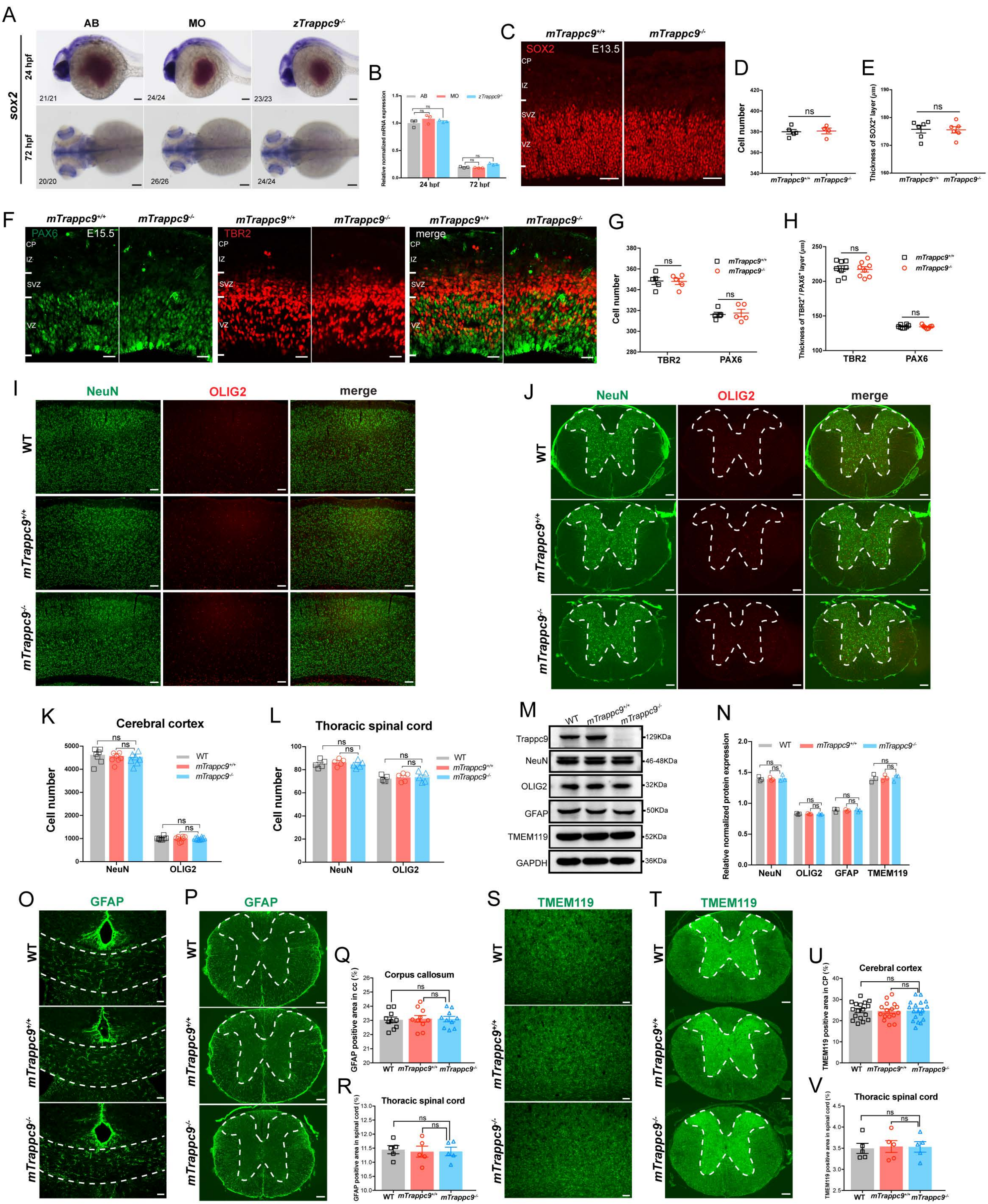
A
AB
MO
zTrappc9-/-
sox2
24 hpf
72 hpf
21/21
24/24
23/23
20/20
26/26
24/24
B
AB
MO
zTrappc9-/-
Relative normalized mRNA expression
24 hpf
72 hpf
C
mTrappc9+/+
mTrappc9-/-
SOX2
E13.5
CP
IZ
SVZ
VZ
D
Cell number
E
Thickness of SOX2+ layer (μm)
F
PAX6
E15.5
TBR2
merge
G
TBR2
PAX6
H
Thickness of TBR2+ / PAX6+ layer (μm)
I
NeuN
OLIG2
merge
WT
J
K
Cerebral cortex
L
Thoracic spinal cord
M
Trappc9
GFAP
TMEM119
GAPDH
129KDa
46-48KDa
32KDa
50KDa
52KDa
36KDa
N
Relative normalized protein expression
O
GFAP
P
Q
Corpus callosum
GFAP positive area in cc (%)
R
GFAP positive area in spinal cord (%)
S
TMEM119
T
U
TMEM119 positive area in CP (%)
V
TMEM119 positive area in spinal cord (%)
ns

Figure S5

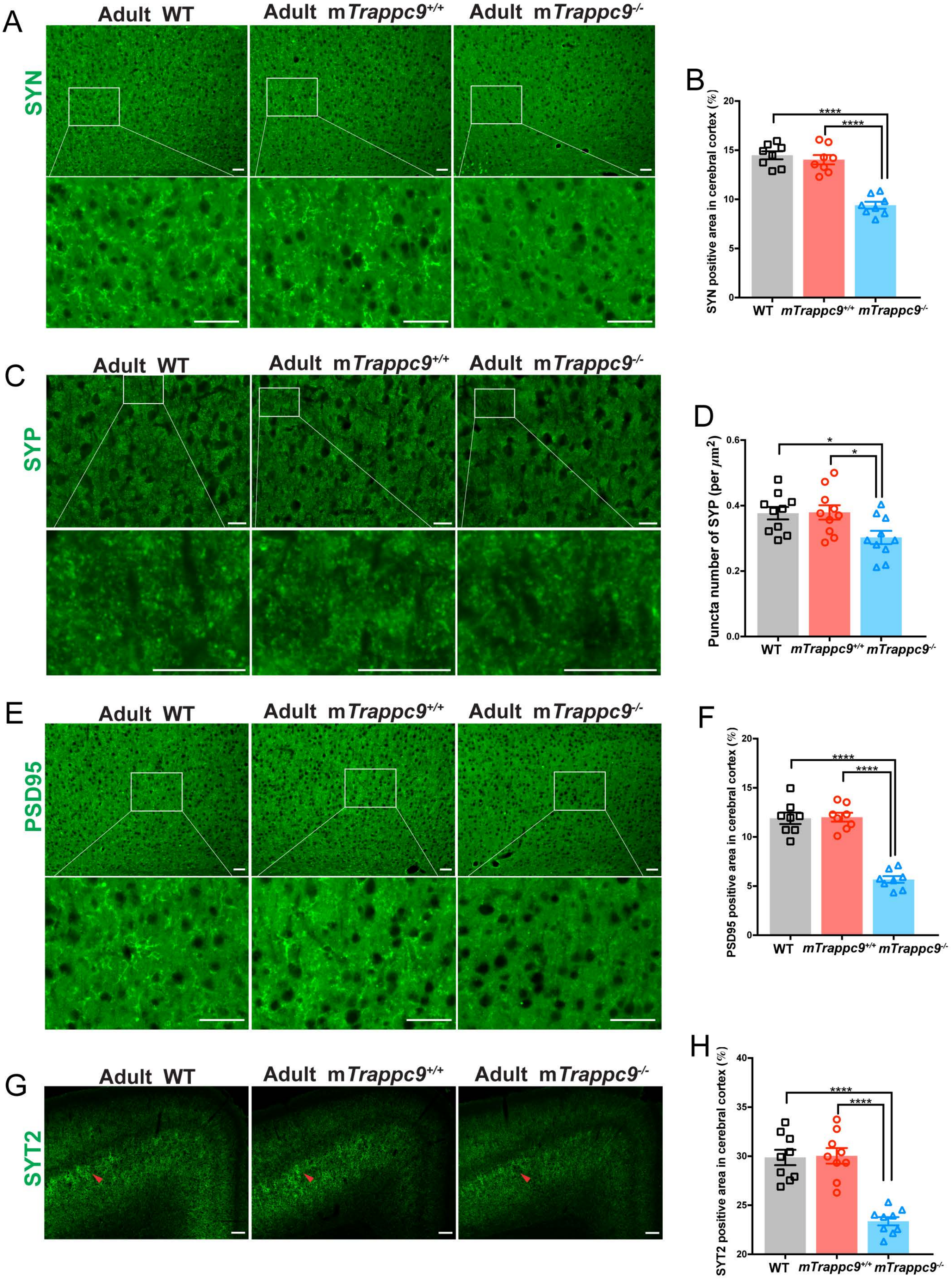
A
Adult WT
Adult mTrappc9+/+
Adult mTrappc9-/-
SYN
B
SYN positive area in cerebral cortex (%)
20
15
10
5
0
****
****
WT
mTrappc9+/+
mTrappc9-/-
C
Adult WT
Adult mTrappc9+/+
Adult mTrappc9-/-
SYP
D
Puncta number of SYP (per μm2)
0.6
0.4
0.2
0.0
*
*
WT
mTrappc9+/+
mTrappc9-/-
E
Adult WT
Adult mTrappc9+/+
Adult mTrappc9-/-
PSD95
F
PSD95 positive area in cerebral cortex (%)
20
15
10
5
0
****
****
WT
mTrappc9+/+
mTrappc9-/-
G
Adult WT
Adult mTrappc9+/+
Adult mTrappc9-/-
SYT2
H
SYT2 positive area in cerebral cortex (%)
40
35
30
25
20
****
****
WT
mTrappc9+/+
mTrappc9-/-

Figure S6

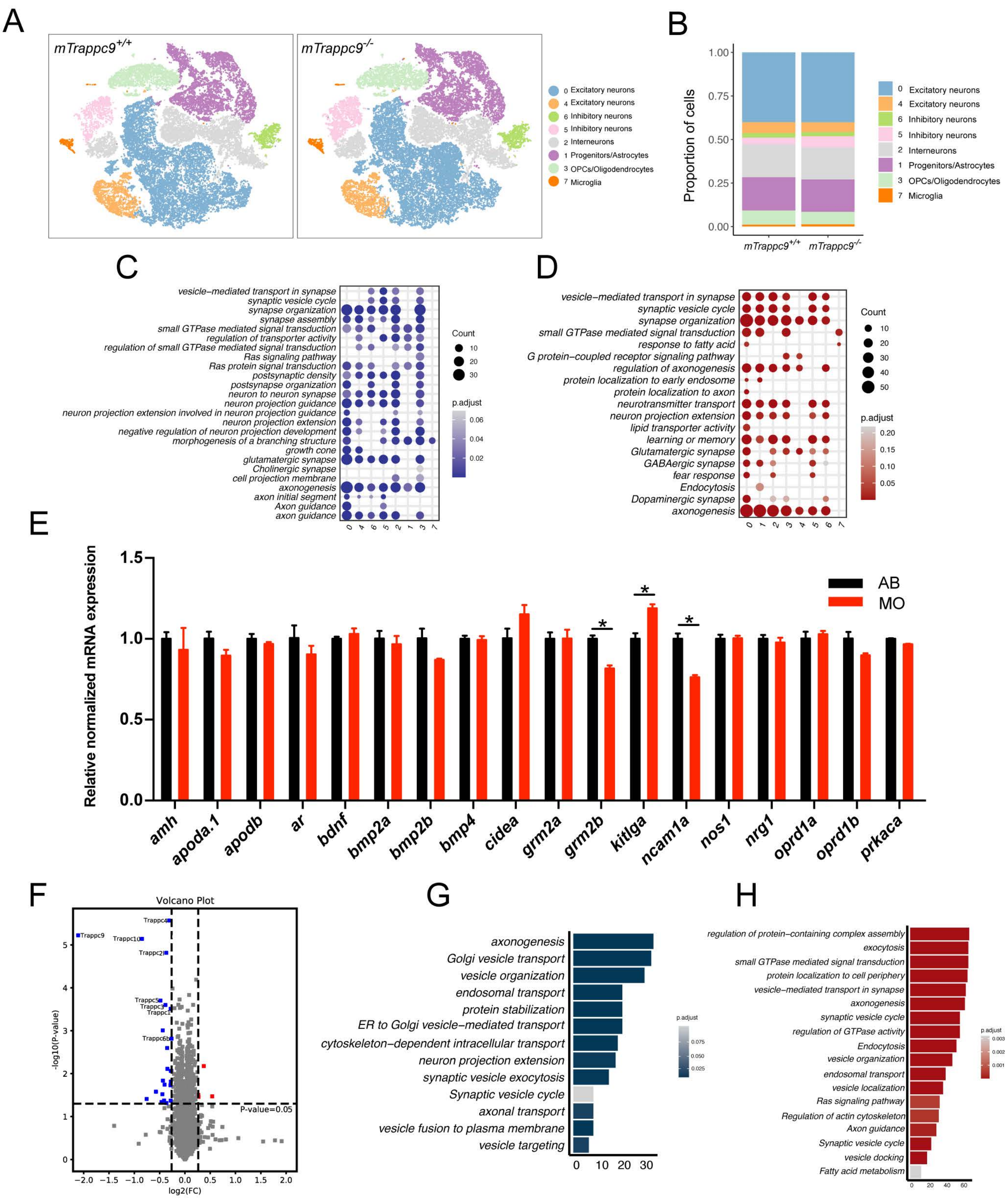

A
mTrappc9+/+
mTrappc9-/-
0 Excitatory neurons
4 Excitatory neurons
6 Inhibitory neurons
5 Inhibitory neurons
2 Interneurons
1 Progenitors/Astrocytes
3 OPCs/Oligodendrocytes
7 Microglia
B
Proportion of cells
C
vesicle–mediated transport in synapse
synaptic vesicle cycle
synapse organization
synapse assembly
small GTPase mediated signal transduction
regulation of transporter activity
regulation of small GTPase mediated signal transduction
Ras signaling pathway
Ras protein signal transduction
postsynaptic density
postsynapse organization
neuron to neuron synapse
neuron projection guidance
neuron projection extension involved in neuron projection guidance
neuron projection extension
negative regulation of neuron projection development
morphogenesis of a branching structure
growth cone
glutamatergic synapse
Cholinergic synapse
cell projection membrane
axonogenesis
axon initial segment
Axon guidance
axon guidance
Count
p.adjust
D
vesicle–mediated transport in synapse
synaptic vesicle cycle
synapse organization
small GTPase mediated signal transduction
response to fatty acid
G protein–coupled receptor signaling pathway
regulation of axonogenesis
protein localization to early endosome
protein localization to axon
neurotransmitter transport
neuron projection extension
lipid transporter activity
learning or memory
Glutamatergic synapse
GABAergic synapse
fear response
Endocytosis
Dopaminergic synapse
axonogenesis
Count
p.adjust
E
Relative normalized mRNA expression
AB
MO
amh
apoda.1
apodb
ar
bdnf
bmp2a
bmp2b
bmp4
cidea
grm2a
grm2b
kitlga
ncam1a
nos1
nrg1
oprd1a
oprd1b
prkaca
F
Volcano Plot
-log10(P-value)
log2(FC)
Trappc9
Trappc4
Trappc10
Trappc2
Trappc5
Trappc3
Trappc1
Trappc6b
P-value=0.05
G
axonogenesis
Golgi vesicle transport
vesicle organization
endosomal transport
protein stabilization
ER to Golgi vesicle–mediated transport
cytoskeleton–dependent intracellular transport
neuron projection extension
synaptic vesicle exocytosis
Synaptic vesicle cycle
axonal transport
vesicle fusion to plasma membrane
vesicle targeting
p.adjust
H
regulation of protein–containing complex assembly
exocytosis
small GTPase mediated signal transduction
protein localization to cell periphery
vesicle–mediated transport in synapse
axonogenesis
synaptic vesicle cycle
regulation of GTPase activity
Endocytosis
vesicle organization
endosomal transport
vesicle localization
Ras signaling pathway
Regulation of actin cytoskeleton
Axon guidance
Synaptic vesicle cycle
vesicle docking
Fatty acid metabolism
p.adjust

Figure S7

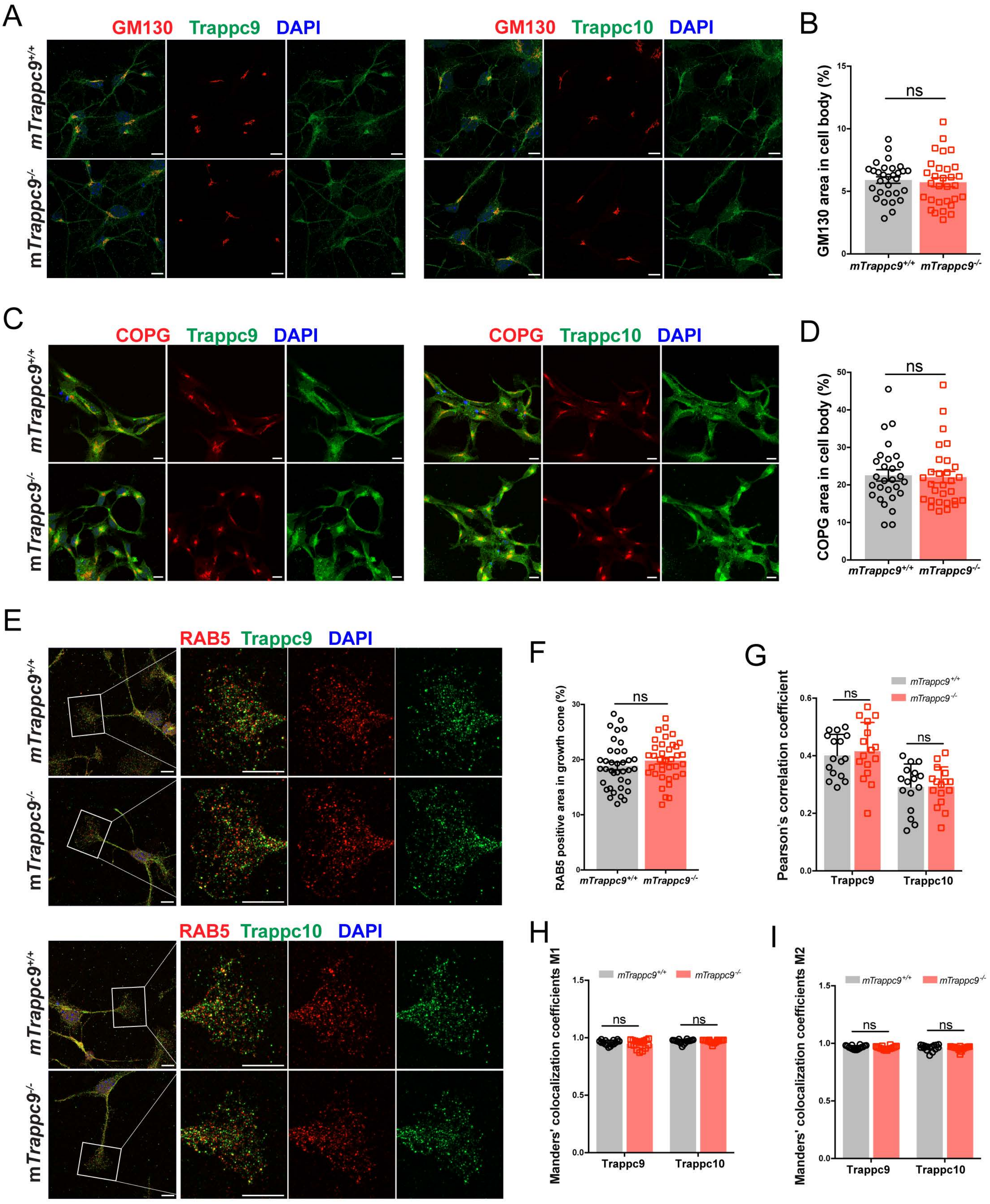
A
GM130 Trappc9 DAPI
GM130 Trappc10 DAPI
mTrappc9+/+
mTrappc9-/-
B
GM130 area in cell body (%)
ns
mTrappc9+/+
mTrappc9-/-
C
COPG Trappc9 DAPI
COPG Trappc10 DAPI
D
COPG area in cell body (%)
E
RAB5 Trappc9 DAPI
RAB5 Trappc10 DAPI
F
RAB5 positive area in growth cone (%)
G
Pearson's correlation coefficient
Trappc9
Trappc10
H
Manders' colocalization coefficients M1
I
Manders' colocalization coefficients M2

Figure S8

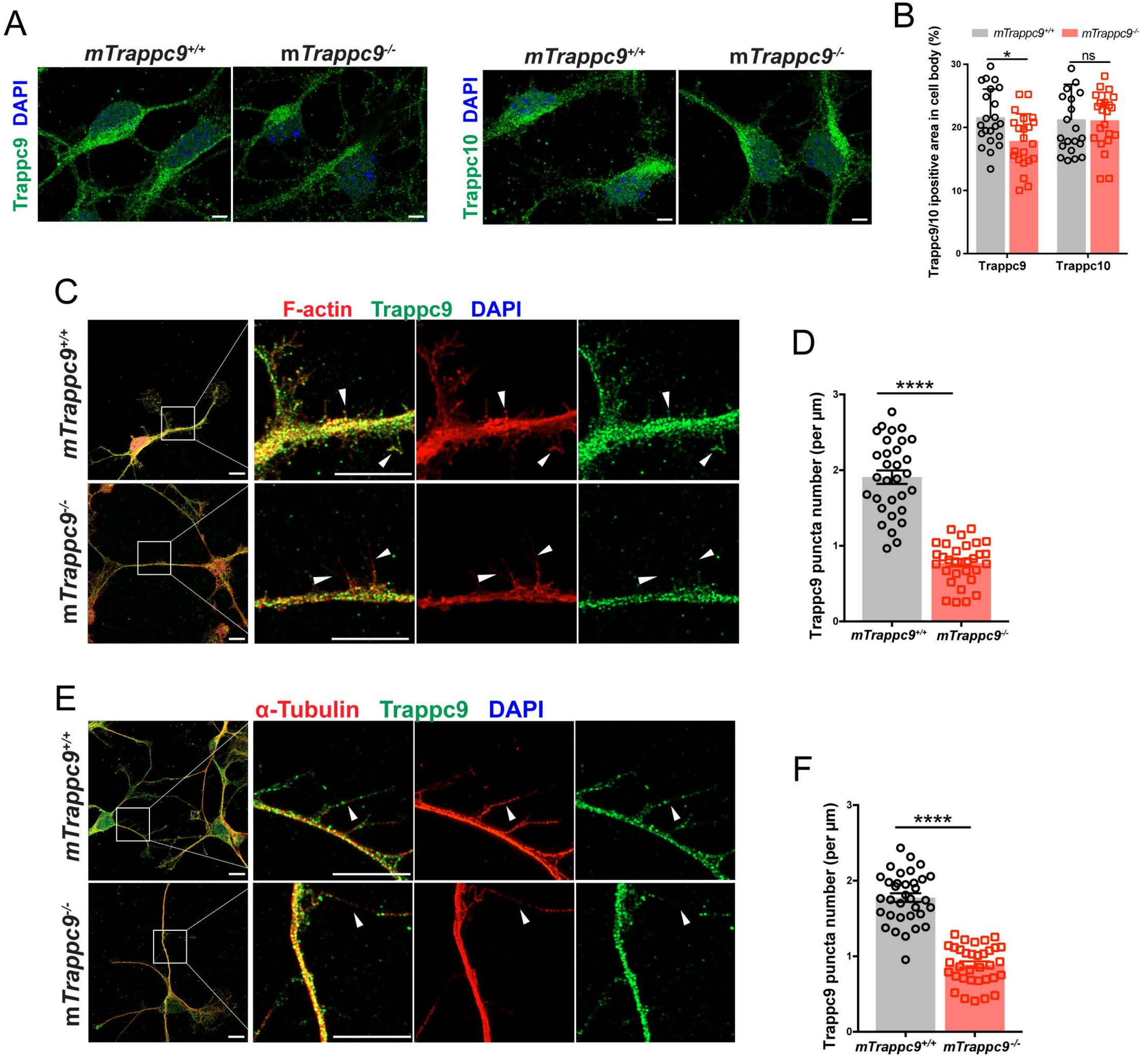

A
mTrappc9+/+
mTrappc9-/-
Trappc9 DAPI
Trappc10 DAPI
B
mTrappc9+/+
mTrappc9-/-
Trappc9/10 ipositive area in cell body (%)
Trappc9
Trappc10
*
ns
C
F-actin Trappc9 DAPI
D
Trappc9 puncta number (per μm)
****
E
α-Tubulin Trappc9 DAPI
F

Figure S9

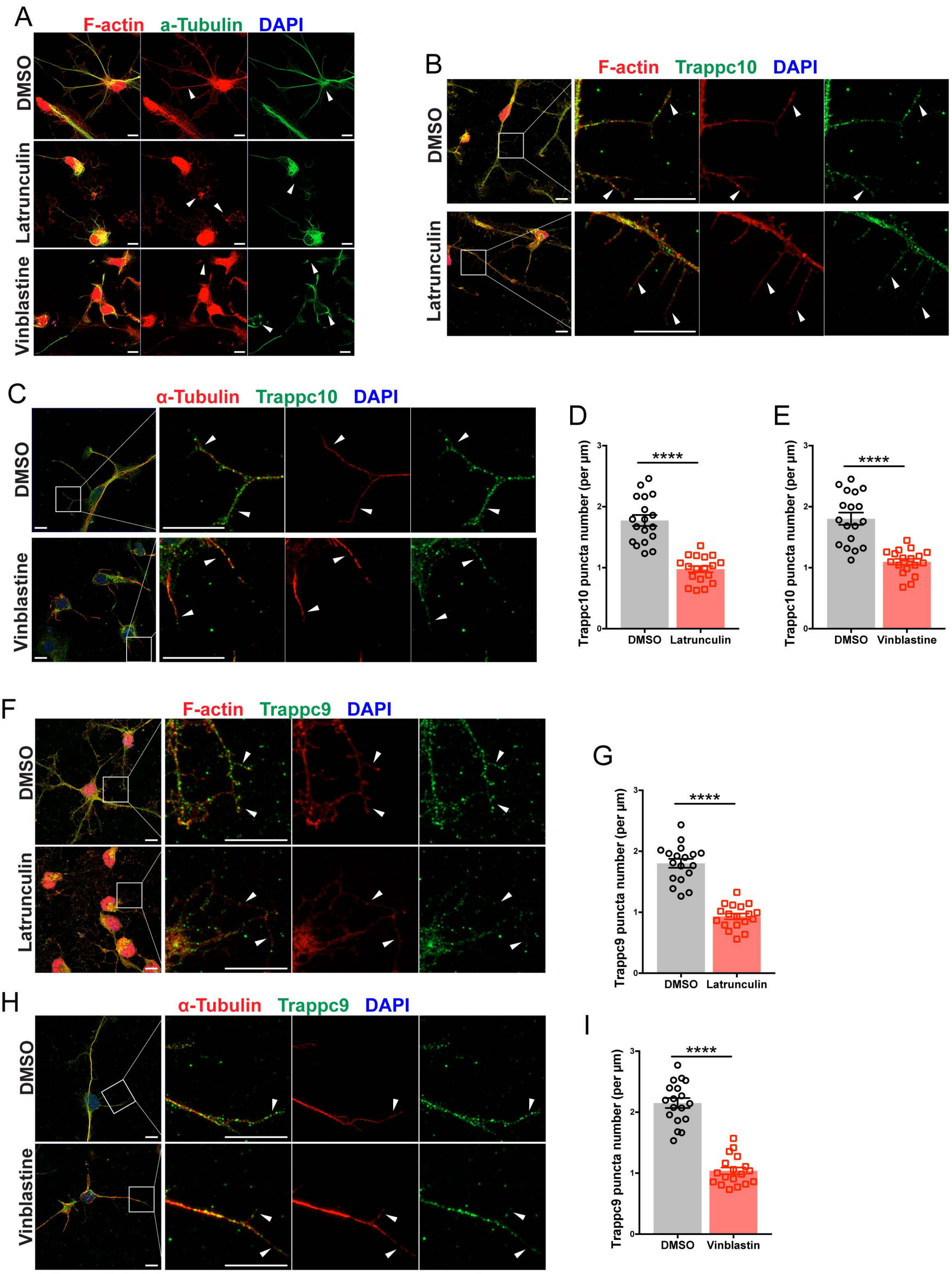
A
F-actin a-Tubulin DAPI
DMSO
Latrunculin
Vinblastine
B
F-actin Trappc10 DAPI
DMSO
Latrunculin
C
α-Tubulin Trappc10 DAPI
DMSO
Vinblastine
D
Trappc10 puncta number (per μm)
****
DMSO Latrunculin
E
Trappc10 puncta number (per μm)
****
DMSO Vinblastine
F
F-actin Trappc9 DAPI
DMSO
Latrunculin
G
Trappc9 puncta number (per μm)
****
DMSO Latrunculin
H
α-Tubulin Trappc9 DAPI
DMSO
Vinblastine
I
Trappc9 puncta number (per μm)
****
DMSO Vinblastin

Figure S10

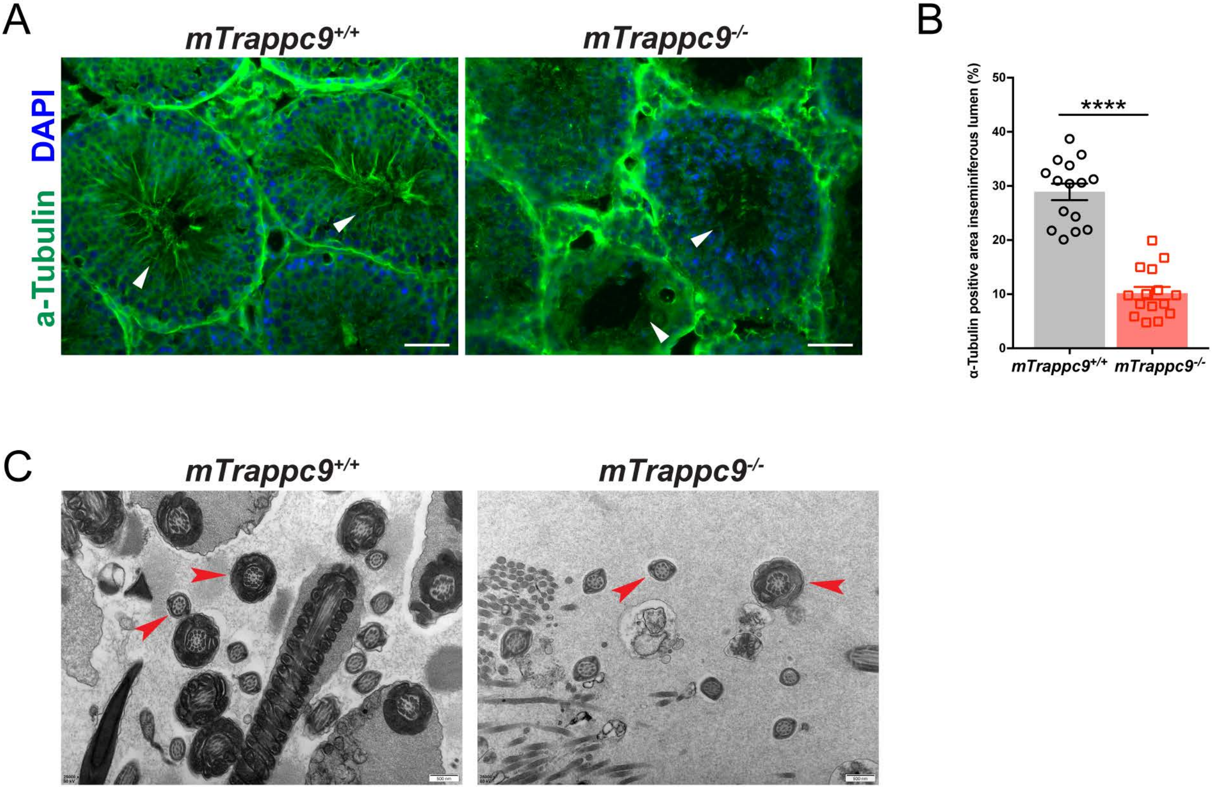

A
mTrappc9+/+
mTrappc9-/-
DAPI
a-Tubulin
B
α-Tubulin positive area inseminiferous lumen (%)
50
40
30
20
10
0
****
mTrappc9+/+
mTrappc9-/-
C
mTrappc9+/+
mTrappc9-/-
